# Study on the influence of COVID-19 on the growth of China’s small and medium-sized construction enterprises

**DOI:** 10.1371/journal.pone.0266315

**Published:** 2022-06-03

**Authors:** Wenbao Wang, Wenhe Lin, Zhenhua Bao, Xinyi Dai, Qiaohua Lin

**Affiliations:** 1 College of Civil Engineering, Yango University, Fuzhou, China; 2 College of Management/College of Tourism, Fujian Agriculture and Forestry University, Fuzhou, China; 3 School of Management, Fujian University of Technology, Fuzhou, China; University of Almeria, SPAIN

## Abstract

The outbreak of COVID-19 at the beginning of 2020 had a significant impact on China’s economy, society, and citizens; it also had a negative impact on the development of the construction industry. In particular, small and medium-sized construction enterprises with low ability to withstand risk have been strongly impacted, aggravating a crisis of survival among these firms. The focus of this study is to analyze the impact of COVID-19 on the growth of small and medium-sized construction companies. Based on the characteristics of small and medium-sized construction enterprises, this paper establishes a growth evaluation index and builds a growth evaluation model based on factor analysis. Twenty-three construction enterprises listed on small and medium-sized enterprises board are selected as samples, and the quarterly data of 2019 and 2020 are used for empirical analysis. The results show that the epidemic has had a high short-term impact on construction enterprises, and the total output value of the construction industry in the first quarter of 2020 was 16% lower than that in the same period of last year. In the long run, the impact of the epidemic on the growth of small and medium-sized construction enterprises has been limited. In the first quarter of 2020, the growth score of enterprises decreased by only 1.95% year-over-year, and it was essentially flat in the second and third quarters. The epidemic has had little influence on solvency, tangible resources or intangible resources but a high short term influence on profitability, capital expansion and market expectations. The long-term impact is small; It is conducive to the improvement of enterprise operation ability. Finally, to both address the influence of the COVID-19 on small and medium-sized construction enterprises and to realize their transformation and upgrading, targeted suggestions are offered at the policy and enterprise levels. The results will help to understand the impact of the epidemic on the growth of construction enterprises, and provide decision support for the healthy and orderly development of the follow-up construction industry.

## 1 Introduction

At the end of 2019, the COVID-19 broke out. According to World Health Organization (WHO) data, by the end of 2020, the number of confirmed cases of COVID-19 in the world had reached 80 million. The infectivity and permeability of COVID-19 were stronger than those of SARS in 2003, and this sudden epidemic has severely threatened people’s safety and disrupted their normal lives. To prevent and control the spread of the epidemic, in January 23, 2020, all cities in China took measures to strictly control travel, delay the resumption of enterprises, suspend social visits and parties, and delay the start of school. The effects of these measures for prevention and control have been remarkable. However, the shutdown of large-scale enterprises and extraordinary nationwide mandatory quarantine measures have also brought high economic costs, affecting all industries to varying degrees [1–3]. In the short term, these strict control measures have had a strong impact on the economy. The direct impact on enterprises has mainly been reflected in reducing the labour participation rate, delaying the resumption of construction and manufacturing, and increasing the labour and other fixed cost burden. Therefore, the transportation, retail, hotel tourism, catering, consumption, warehousing, postal, real estate and construction industries have all been affected to some extent. The construction industry is labour intensive, has more open-air operations, and must transfer production sites to the location of construction projects. Compared with the traditional manufacturing industry, standardized management is difficult, and epidemic prevention and control conditions are more complex. At present, small and medium-sized construction enterprises account for more than 90% of all construction enterprises in China [4], making them an indispensable part of China’s construction industry. Therefore, it is important to study the impact of COVID-19 on the growth of small and medium-sized construction enterprises.

Current research on the influence of the COVID-19 mainly focuses on the following areas. One stream of research studies the influence of the COVID-19 on economic growth, foreign trade, employment, production and consumption, for example, in terms of economy, studies on the impact of COVID-19 on the economy and relevant measures taken by various countries to solve the problem [5, 6]. Studies show that the epidemic has a huge impact on the world economy and will cause a loss of more than 2.2 billion US dollars to the world GDP by the end of 2020 [7, 8]. Countermeasures and suggestions are proposed based on the characteristics of various industries from the policy, economic and financial levels [9, 10]. In terms of trade, studies show that COVID-19 has a certain negative impact on import and export trade, but the impact is only a short-term fluctuation [11]. It is suggested to take corresponding measures in policy response to rapidly recover trade development and reduce dependence on foreign investment [12]. In terms of employment, COVID-19 has a short-term impact on employment and unemployment, but the impact is large [13, 14], especially in big cities [15]. The epidemic has also had a negative impact on production and consumption [16, 17], and "compensatory consumption" after the epidemic can mitigate the impact on annual consumption growth to a certain extent. At the same time, research [18] shows that fear appeal (fears for health and economic fears) are associated with the changes in customer behaviour and influence traditional and online shopping related to COVID-19. The other stream of research studies the difficulties faced in the development of tourism, agriculture, transportation, and the timber industry under the influence of COVID-19. For example, to study the mechanism of COVID-19’s impact on tourism, [19, 20] points out that private and public policy support must be coordinated to sustain pre-COVID-19 operational levels of the tourism and travel sector [21, 22]. In agriculture, studies have shown that [23, 24] COVID-19 has produced certain effects on food security and agricultural development. COVID-19 has obvious impact on the production and business activities of wood industry, and there are some differences in the impact on different types of wood enterprises [25]. At the same time, the epidemic also has a great impact on the transportation industry, and the unemployment rate of workers in the transportation industry is higher than that in other industries [26, 27].

Like the above industries, the epidemic has also had a certain impact on the construction industry. COVID-19 has a great impact on the construction period, cost and the market, business, operation, personnel turnover and staff working status of [28–31]. It reduces the financial performance of construction enterprises [32], affects the [33] of building stakeholders, and the government and enterprises should complete emergency mechanisms for major public emergencies. Sharing emergency management data and other strategies to deal with COVID-19’s impact [34]. In addition, epidemic factors have also greatly increased legal disputes over construction contracts. It should be determined whether the epidemic is an event of force majeure in the construction contract in combination with individual cases and parties’ claims [35, 36]. Most of the above studies qualitatively analyzed the impact of the epidemic on construction enterprises from the aspects of shutdown, labor and raw materials, construction period, cost, quality and safety. However, few studies have explored the influence of COVID-19 on the growth of small and medium-sized construction enterprises. Therefore, it is important to explore the influence of the epidemic on the growth of small and medium-sized construction enterprises based on the financial quarterly reports of listed companies, as the research results can provide decision support for the healthy and orderly future development of the construction industry.

## 2 Research methods and evaluation indicators

### 2.1 Research methods

An analysis and summary of the literature find that the commonly used methods for evaluating enterprise growth mainly include the factor analysis method, principal component analysis method, catastrophe progression method, grey correlation method, analytic hierarchy process, entropy TOPSIS method, and entropy VIKOR method [37–40]. By analyzing the internal relationship of events, factor analysis method grasps the main contradictions and finds out the main factors, which makes the multivariable complex problems easy to study and analyze [41]. Factor analysis method has the characteristics of finding a few comprehensive factors from multiple observation variables to explain the original data, which is helpful to objectively and effectively determine the weight of comprehensive indicators, and has good objectivity. It can fundamentally remove the influence of the correlation between indicators, and is suitable for the comprehensive evaluation of the object system with correlation between evaluation indicators [42]. Factor analysis also has shortcomings. For example, the weight is based on data analysis and is not affected by subjective factors, so it cannot reflect the subjective importance of indicators. The evaluation result is related to the sample size, and it assumes that the relationship between indicators is linear. Although the factor analysis method still has some shortcomings, it is undeniable that it plays an important role in comprehensive evaluation. There are many studies using the factor analysis method, such as Lu Tao and Pan Li [43], who used factor analysis to evaluate the growth of 36 new energy listed companies, and Quan Liang et al. [44] evaluated the growth of 30 listed forestry companies by factor analysis. Combined with the characteristics of factor analysis and the objectivity of sample data, this study selects factor analysis for evaluation, and uses spss21.0 as an analysis tool for statistical analysis of sample data.

The model of this study mainly applies factor analysis, including standardized processing of index data, model building, factor load, factor rotation, factor score, etc. The specific methods are as follows:

(1) Standardized processing of index data

Considering that each indicator has different dimensions, it is necessary to perform dimensionless processing on the data [45].

When the evaluation index meets the criterion to be classified as the benefit type (the larger the value, the better), *x*_*ij*_ is calculated as follows:

$$x_{ij}=\frac{x_{ij}^{\prime }-\underset{i}{\text{min}}\left(x_{ij}^{\prime }\right)}{\underset{i}{\text{max}}\left(x_{ij}^{\prime }\right)-\underset{i}{\text{min}}\left(x_{ij}^{\prime }\right)}$$ (1)

When the evaluation indicator is among the cost type criteria (the smaller the value, the better), *x*_*ij*_ is calculated as follows:

$$x_{ij}=\frac{\underset{i}{\text{max}}\left(x_{ij}^{\prime }\right)-x_{ij}^{\prime }}{\underset{i}{\text{max}}\left(x_{ij}^{\prime }\right)-\underset{i}{\text{min}}\left(x_{ij}^{\prime }\right)}$$ (2)

When the indicator is a fixed criterion (the closer the value is to an ideal value *β*, the better), *x*_*ij*_ is calculated as follows:

$$x_{ij}=1.0-\left|x_{ij}^{\prime }-\beta \right|/\underset{i}{\text{max}}\left|x_{ij}^{\prime }-\beta \right|$$ (3)

(2) Establish a factor analysis model

$$\left\{\begin{array}{c} X_{1}=a_{11}F_{1}+a_{12}F_{2}+\cdots +a_{1m}F_{m}+\xi_{1}\\ X_{2}=a_{21}F_{1}+a_{22}F_{2}+\cdots +a_{2m}F_{m}+\xi_{2}\\ \vdots \\ X_{n}=a_{n1}F_{1}+a_{n2}F_{2}+\cdots +a_{nm}F_{m}+\xi_{n} \end{array}\right.$$ (4)

(3) Determine whether factor analysis is applicable

Bartlett test and KMO test are generally used to determine whether the original data comply with multivariate normal distribution and further determine whether the sample data have the conditions for factor analysis. Bartlett sphericity test is a statistical method established by using the determinant of correlation matrix. If the corresponding concomitant probability value is less than the significance level of 0.05, factor analysis is suitable [46]. KMO test is used to measure the partial correlation of variables. KMO > 0.5, factor analysis is suitable; KMO < 0.5, factor analysis is not suitable [47].

(4) Extraction of common factors

After extracting the principal components by spss20.0, the variance contribution rate, initial eigenvalue and cumulative contribution rate of each factor are obtained. The variance contribution rate reflects the contribution degree of each factor, and the cumulative contribution rate reflects the original information of the principal component. The common judgment standard for selecting the number of main factors is to take the number of main factors with eigenvalue greater than 1 or cumulative contribution rate of more than a certain value (60% [46], 80% [48]). In this study, the number of factors with eigenvalue greater than 1 or cumulative contribution rate of 70% is taken.

(5) Factor rotation and naming

In order to obtain more explicit analysis results, the factor load matrix is often rotated, so that the relationship between the original variables is easier to interpret the information after redistribution, so as to name the factors. In this study, the maximum variance method is used to rotate the factor load matrix orthogonally.

(6) Factor score calculation

Calculate the score of each factor according to the factor score coefficient table:

$$F_{j}=\alpha_{j1}X_{1}+\alpha_{j2}X_{2}+\cdots +\alpha_{jp}X_{p},j=1,2,\cdots ,m$$ (5)

The comprehensive score is calculated according to the score of a single factor and the weight of each common factor:

$$F=w_{1}F_{1}+w_{2}F_{2}+\cdots +w_{j}F_{j},j=1,2,\cdots ,m$$ (6)

Where *m* is the number of common factors, *f*_*j*_ is a single factor score, *w*_*j*_ is the variance contribution rate of the *j*-th common factor.

### 2.2 Selection of evaluation indicators

The selection of the evaluation index directly influences whether the resulting index system can be effectively applied in the evaluation process. The China Enterprise Evaluation Association has proposed the GEP evaluation method, which uses quantitative financial indicators such as the sales revenue growth rate, net profit growth rate, net asset growth rate, total asset return rate, capital return rate and asset-liability ratio combined with qualitative indicators to form a system evaluating the growth of small and medium-sized enterprises [49]. In creating an index, this paper considered the principle that selected indexes should be comprehensive, incorporated Penrose’s enterprise growth theory [50] and referred to the research results of Chinese scholars in recent years [51–53]. The characteristics of listed construction enterprises were considered, such as the coexistence of economy and profitability, labour intensity, high resource consumption, sensitivity to policy, impacts on the ecological environment, as well as the characteristics of profitability and operation, development and expansion, debt repayment and risk resistance [54]. Incorporating all these factors, a growth evaluation index system of small and medium-sized construction enterprises was created (as shown in Table 1). The indicator system consists of a total of 20 indicators.

**Table 1:** The growth evaluation index system of small and medium-sized construction enterprises.

Target Layer	Indicator Layer
The growth evaluation index system of small and medium-sized construction enterprises	Ratio of intangible assets X_1_, Growth rate of intangible assets X_2_
	Increasing rate of fixed assets X_3_, Cash ratio X_4_, Inventory X_5_, Accounts receivable X_6_
	Number of employees X_7_
	Current asset turnover X_8_, Turnover of fixed assets X_9,_ Inventory turnover X_10,_ Accounts receivable turnover X_11_
	Operating gross profit margin X_12_, Return on equity X_13_, Return on assets X_14_
	Asset-liability ratio X_15_, Current ratio X_16_, Quick ratio X_17_
	Net profit margin on sales X_18_
	Price earning (PE) ratio X_19_, Price/book (PB) value ratio X_20_

### 2.3 Sample selection and data source

By the end of 2020, the COVID-19 has been effectively controlled in China, and the impact of the entire epidemic chain on the growth of SMEs, including the initial, intermediate and later stages of the epidemic, can be seen. Therefore, it is representative to select Chinese enterprises as research objects. According to the division of enterprise scale in China, small and medium-sized construction enterprises mainly refer to construction enterprises with less than 3000 employees, annual output value less than 300 million yuan, or total assets less than 400 million yuan [55]. For data collectability, This study selects the construction enterprises listed on the SME Board of Shenzhen Stock Exchange as the sample, and according to the growth evaluation index system, the quarterly data of 2019 and 2020 are collected and sorted for analysis. Combined with the 20 indicators selected as conditions, the enterprises with missing indicators and incomplete financial data are eliminated, and 23 enterprises are ultimately selected. The samples are shown in Table 2. The index data come from the information disclosed in the quarterly reports of the sample enterprises obtained from the Wind database.

**Table 2:** The sample list.

Number	Stock code	COHR	Number	Stock code	COHR
1	002047	BAUING	14	002375	YASHA
2	002051	CAMC	15	002431	PALM
3	002060	GHE	16	002469	Sanwei
4	002061	ZCT	17	002482	GRANDLAND
5	002062	HR	18	002541	HOLU
6	002081	Gold Mantis	19	002542	ZHCGE
7	002116	China Haisum	20	002545	QDDFTT
8	002135	ZSSF	21	002620	Ruihe
9	002140	DHC	22	002628	CDR&B
10	002178	Yanhua Smartech	23	002663	Pbland
11	002307	BXLQ	24		
12	002310	Orient Landscape	25		
13	002325	HONGTAO	26		

## 3 Empirical research

The collected data were processed through the following steps.

### (1) Standardize the data

After obtaining the data, these indicators are standardized first. Among the 20 indicators selected in this study, inventory, accounts receivable root and P / E ratio are cost indicators, which are standardized according to formula (2); Asset liability ratio, current ratio, quick ratio and book value ratio are fixed indicators, which are standardized according to formula (3); Other indicators are benefit indicators, and the data can be standardized according to formula (1).

### (2) Applicability test of factor analysis

KMO and Bartlett tests were performed on the data, as shown in Table 3. The KMO value of the sample data is 0.575, which is greater than 0.5 and passes the KMO test. The observed value of the Bartlett sphericity test statistic is 1805.953, and the concomitant probability of the statistic is 0.000, which indicates that there is a significant difference between the correlation coefficient matrix and the identity matrix. Therefore, the sample data are suitable for factor analysis.

**Table 3:** Test table of KMO and Bartlett.

Items		Test value
Kaiser-Meyer-Olkin measure of sampling adequacy		0.575
Bartlett sphericity test	Approximate chi square	1805.953
	df	190
	Sig.	0.000

### (3) Number of determining factors

According to the principle of an eigenvalue greater than 1, this paper selects seven common factors, as shown in Table 4, that can explain 73.048% (>70%) of the total variance of the original variable. According to factor analysis theory, the main factors should reflect more than 60% of the variance variation, therefore, the number of principal factors extracted in this study is reasonable.

**Table 4:** Eigenvalues and contribution rate of main factors.

Factors	Eigenvalues	Contribution rate (%)	Cumulative contribution rate (%)
1	3.620	18.100	18.100
2	3.130	15.649	33.749
3	2.259	11.293	45.042
4	1.818	9.088	54.130
5	1.451	7.255	61.385
6	1.195	5.975	67.361
7	1.138	5.688	73.048

### (4) Factor load matrix after rotation

To make the naming of common factors and the interpretation of variables more practical, this study uses the maximum variance method to orthogonally rotate the factor load matrix, as shown in Table 5. According to the principle of naming those factors with a larger load on each common factor, the common factors F1, F2, F3, F4, F5, F6 and F7 are named profitability, solvency, tangible resources, intangible resources, operation ability, capital expansion ability and market expectation ability, respectively.

**Table 5:** The factor load table after rotation.

Index	Principal factor						
	1	2	3	4	5	6	7
X_1_	-0.015	0.178	-0.190	0.791	-0.133	-0.136	0.026
X_2_	-0.062	0.021	-0.080	-0.008	0.089	0.509	-0.114
X_3_	0.211	-0.071	0.020	-0.109	-0.167	0.546	0.039
X_4_	0.071	-0.754	-0.098	0.345	0.328	0.038	0.093
X_5_	0.006	0.002	0.050	0.217	0.783	0.217	0.067
X_6_	0.051	-0.082	-0.901	0.000	0.152	-0.182	0.064
X_7_	0.238	0.108	0.769	-0.159	-0.062	-0.139	0.050
X_8_	0.823	0.090	-0.008	-0.177	-0.143	0.132	0.233
X_9_	0.089	0.075	0.167	-0.042	0.087	0.794	0.084
X_10_	0.129	-0.002	0.766	0.009	0.150	0.020	0.066
X_11_	0.719	0.058	-0.327	-0.278	-0.271	-0.069	0.176
X_12_	-0.037	-0.398	0.191	0.660	-0.095	-0.070	-0.295
X_13_	0.873	-0.047	0.293	0.000	0.152	0.039	-0.087
X_14_	0.822	-0.098	0.329	0.101	0.054	0.130	-0.100
X_15_	-0.077	-0.190	-0.066	0.806	0.254	-0.038	0.263
X_16_	-0.055	0.868	0.148	0.196	0.127	0.092	0.182
X_17_	0.044	0.963	-0.010	-0.098	-0.063	-0.023	-0.051
X_18_	0.619	-0.088	0.051	0.313	0.372	0.034	-0.318
X_19_	0.007	-0.105	-0.075	-0.281	0.611	-0.157	-0.057
X_20_	0.018	0.029	0.053	0.081	0.004	-0.035	0.888

### (5) Score calculation

In this paper, the score coefficient is estimated by stepwise regression analysis, as shown in Table 6. According to formula (5), the Calculated functions of different factor scores can be obtained. For example, the scoring function of the first factor F1 is:

$${\text{F}}_{1}=0.052{\text{X}}_{1}-0.048{\text{X}}_{2}+0.031{\text{X}}_{3}+\cdots -0.002{\text{X}}_{20}$$

**Table 6:** Factor score coefficient matrix.

Index	Principal factor						
	1	2	3	4	5	6	7
X_1_	0.052	0.140	-0.086	0.412	-0.160	-0.047	-0.014
X_2_	-0.048	0.015	-0.075	0.010	0.024	0.393	-0.107
X_3_	0.031	-0.063	-0.038	-0.009	-0.158	0.412	0.026
X_4_	0.017	-0.273	-0.025	0.070	0.128	0.034	0.125
X_5_	-0.018	0.070	-0.008	0.019	0.507	0.106	0.056
X_6_	0.089	0.015	-0.383	-0.022	0.130	-0.087	0.029
X_7_	0.032	0.001	0.331	-0.069	-0.019	-0.184	0.065
X_8_	0.266	0.013	-0.063	-0.030	-0.091	0.046	0.176
X_9_	-0.034	0.008	0.007	0.009	0.004	0.577	0.056
X_10_	-0.015	-0.023	0.326	-0.019	0.089	-0.063	0.084
X_11_	0.264	0.000	-0.178	-0.075	-0.149	-0.069	0.124
X_12_	-0.003	-0.110	0.091	0.300	-0.178	-0.015	-0.218
X_13_	0.270	0.007	0.059	0.010	0.083	-0.058	-0.066
X_14_	0.248	-0.018	0.074	0.068	-0.010	0.026	-0.076
X_15_	0.000	-0.013	-0.016	0.338	0.074	-0.006	0.217
X_16_	-0.010	0.378	0.028	0.149	0.128	0.039	0.087
X_17_	0.039	0.408	-0.047	0.041	0.045	-0.038	-0.115
X_18_	0.212	0.054	-0.043	0.131	0.202	-0.027	-0.264
X_19_	-0.003	-0.009	-0.026	-0.224	0.469	-0.174	-0.027
X_20_	-0.002	-0.049	0.057	0.022	0.006	-0.046	0.733

Similarly, the factor scores of F2, F3, F4, F5, F6 and F7 are calculated. The weight of each factor is determined according to the total variance data explained in Table 4, and the calculation method is the variance contribution rate/cumulative variance contribution rate of each factor. The weights of common factors F1, F2, F3, F4, F5, F6 and F7 are 0.248, 0.214, 0.155, 0.124, 0.099, 0.082 and 0.078, respectively. According to formula (6),, the comprehensive score function of enterprise growth is obtained

$$\text{Growth}=0.248{\text{F}}_{1}+0.214{\text{F}}_{2}+0.155{\text{F}}_{3}+0.124{\text{F}}_{4}+0.099{\text{F}}_{5}+0.082{\text{F}}_{6}+0.078{\text{F}}_{7}$$

The growth scores for each quarter are shown in Table 7.

**Table 7:** Comprehensive score of enterprises in each quarter.

COHR	2019				2020		
	First quarter	Second quarter	Third quarter	Fourth quarter	First quarter	Second quarter	Third quarter
BAUING	0.482	0.516	0.550	0.560	0.460	0.496	0.518
CAMC	0.476	0.490	0.502	0.540	0.454	0.474	0.482
GHE	0.407	0.429	0.449	0.474	0.422	0.439	0.456
ZCT	0.405	0.430	0.456	0.485	0.440	0.478	0.503
HR	0.397	0.413	0.446	0.481	0.425	0.443	0.467
Gold Mantis	0.544	0.581	0.632	0.664	0.515	0.565	0.617
China Haisum	0.461	0.493	0.523	0.529	0.448	0.439	0.476
ZSSF	0.456	0.477	0.496	0.513	0.463	0.489	0.516
DHC	0.444	0.462	0.483	0.516	0.445	0.464	0.484
Yanhua Smartech	0.460	0.475	0.488	0.494	0.441	0.464	0.483
BXLQ	0.382	0.400	0.416	0.445	0.374	0.442	0.454
Orient Landscape	0.347	0.325	0.346	0.369	0.395	0.446	0.430
HONGTAO	0.471	0.483	0.500	0.508	0.449	0.459	0.480
YASHA	0.475	0.487	0.503	0.518	0.460	0.474	0.486
PALM	0.369	0.378	0.388	0.313	0.347	0.426	0.449
Sanwei	0.354	0.366	0.354	0.372	0.255	0.344	0.358
GRANDLAND	0.481	0.518	0.556	0.545	0.453	0.458	0.498
HOLU	0.472	0.493	0.530	0.565	0.449	0.494	0.532
ZHCGE	0.473	0.493	0.509	0.528	0.474	0.479	0.505
QDDFTT	0.524	0.567	0.573	0.595	0.530	0.555	0.568
Ruihe	0.471	0.492	0.512	0.526	0.435	0.474	0.496
CDR&B	0.449	0.468	0.484	0.497	0.450	0.470	0.485
Pbland	0.473	0.503	0.515	0.420	0.489	0.493	0.519

## 4. Empirical research results and analysis

Literature [56] points out that the short-term impact of the COVID-19 on domestic economic development is mainly concentrated in the first quarter of the year. Traditional manufacturing and service industries requiring producers or consumers to visit the site are more vulnerable to the impact of the COVID-19, such as construction industry. Literature [57] shows that the operation of small and medium-sized listed enterprises in Hubei is very poor in the first quarter of 2020. These research results are consistent with the research results of this paper (the average growth rate of enterprise growth in the first quarter of 2020 is -12.11%), which shows the rationality of the research model.

**Fig 1:**
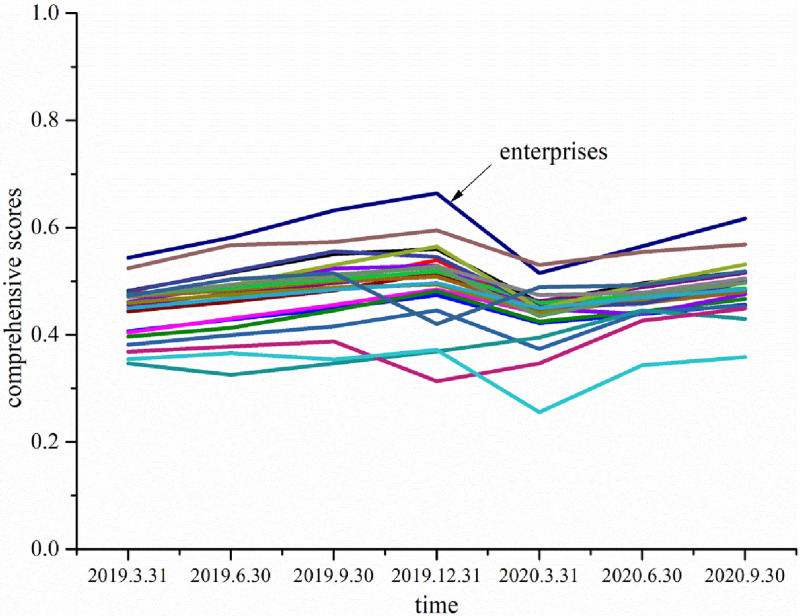
Comprehensive score of enterprises in each quarter.

As seen in Table 7, the growth trend of most enterprises is an improvement from the first quarter to the fourth quarter of 2019 (the average growth rates of the second, third and fourth quarters is 4.55%, 4.40% and 2.21%, respectively), while the first to third quarters of 2020 show a fluctuating trend (the average growth rate of the first, second and third quarters is -12.11%, 6.86% and 4.64%). Growth scores declined in the first quarter of 2020 (down 1.95% year-over-year, on average) and were almost flat in the second and third quarters compared to 2019 (up 0.22% and 0.45% year-over-year, on average), as shown in Fig 1. According to the data from the National Bureau of Statistics of China, the GDP in the first quarter of 2020 was 206.5043 trillion yuan (2.91463 trillion dollars), down 5.3% year-over-year. In the first quarter of 2020, the total output value of the construction industry was 3,591,659 billion yuan (506.93 billion dollars) down 16% year-over-year; in the second quarter of 2020, the total output value of the construction industry was 6,492,353 billion yuan (917.06 billion dollars), up 10.31% year-over-year; and in the third quarter of 2020, the total output value of the construction industry was 6,708,688 billion yuan (985.11 billion dollars), up 10.35% year-over-year). This shows that the short-term impact of the epidemic on construction companies was high, but the long-term impact has been limited. The high short-term impact was mainly due to the delayed resumption of work, restrictions on the return of migrant workers, policy requirements for local re-employment, manpower shortages, unfavourable transportation of materials and equipment, a long process of approval for resumed projects and other adverse factors caused by COVID-19 that delayed construction schedules and affected the contract period [31, 34]. The cost of site shutdown, slow construction and relevant epidemic prevention and control measures resulted in capital waste and cost increases, and the cost of raw materials, labour and transportation has risen [58], which greatly affected the output value and income of the construction industry in the first quarter. The short-term impact on construction enterprises is greater. In terms of long-term impact, the first quarter is the traditional off-season for construction due to factors such as the Spring Festival and the limited construction conditions in winter, so the affected projects can catch up by taking relevant measures in later periods. At the same time, the research shows that the impact of the epidemic on industrial development will show industry heterogeneity [56]. Compared with other industries, the business cycle of the construction industry is longer, and the projects affected by the epidemic can be made up through the rush period in the later stage. Therefore, in the first quarter of 2020, the year-over-year decrease in the enterprise growth score was only 1.95%, while in the second and third quarters, the year-over-year decrease was basically flat. The long term influence of the epidemic on the growth of small and medium-sized construction enterprises has been limited.

**Fig 2:**
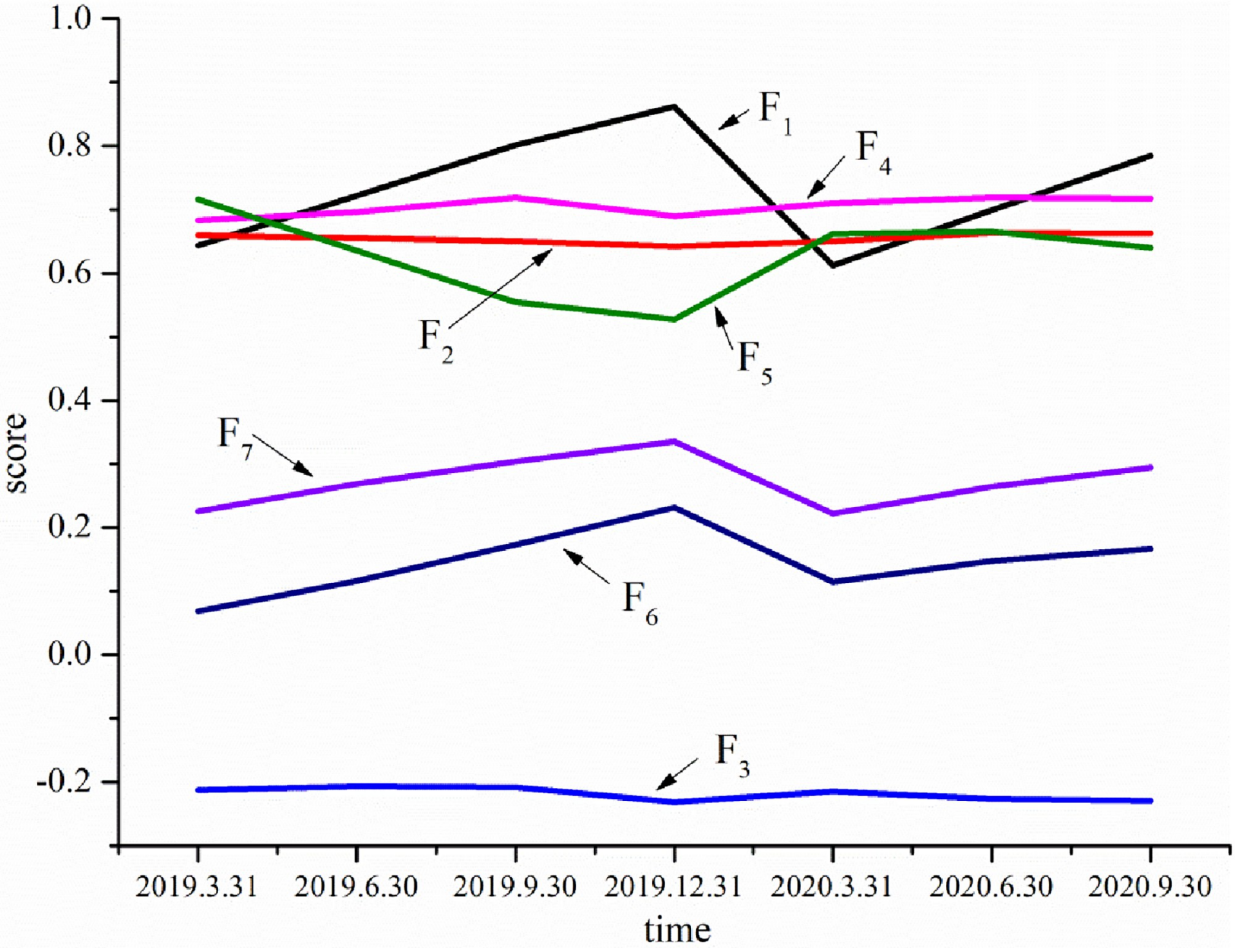
The quarterly average score of common factors.

Further analysis was performed on the quarterly score trend for various factors of enterprises. As shown in Fig 2, the average scores of common factors F2, F3 and F4 changed little in each quarter in 2020, indicating that the epidemic had little influence on debt paying ability, tangible resources or intangible resources. This is because the government has increased financial support for small and medium-sized enterprises, introduced policies such as credit support and loan risk compensation [57] to ensure the solvency of enterprises. At the same time, tangible and intangible resources are not affected in the short term. The average scores of common factors F1 (profitability), F6 (capital expansion ability) and F7 (market expectation ability) decreased by 29%, 50.67% and 33.80% respectively in the first quarter of 2020. The average scores in the second and third quarters increased slightly, indicating that the epidemic has a great short-term impact on common factors F1, F6 and F7, but little long-term impact Due to the impact of the epidemic, prevention and control measures such as rework, isolation of personnel, procurement of epidemic prevention materials, and enhancement of daily monitoring and control led to an increase in costs [34]. After the end of the epidemic, centralized construction caused a shortage of building materials, machinery and equipment, and the "labour shortage" increased project costs. During the epidemic, governments at all levels focused on epidemic prevention and control. The progress of bidding and contracting new projects was affected to a certain extent, related construction investment was delayed, and small and medium-sized construction enterprises lacked short-term funds. These have affected the development of new projects and the smooth progress of old projects, so that the common factors F1, F6 and F7 have a great impact in the short term. With the implementation of relevant policies and the effective control of the COVID-19, the impact of the COVID-19 on common factors F1, F6 and F7 is reduced, and the long-term impact is small, which shows that the relevant policies of the construction industry play a positive role. The average score of common factor F5 (operating capacity) showed an upward trend in 2020, especially 25.5% in the first quarter of 2020, indicating that the impact of the epidemic prompted enterprises to strengthen their own capacity-building, improve the application level of intelligence and informatization, improve the ability of emergency prevention and control and risk management, and accelerate industrial optimization and upgrading.

## 5 Conclusions and suggestions

### 5.1 Conclusions

COVID-19 occurred before the Spring Festival in 2020. The post-holiday labour shortage in the construction industry and the delayed resumption of work caused by the epidemic caused a significant decline in the resumption of work and slow progress in construction projects, forcing construction enterprises to bear a more uncertain payment cycle and greater risk of default. The lack of cash reserves after the Spring Festival and the rise in construction costs caused by the epidemic mean that many small and medium-sized construction enterprises are facing difficulties such as breaches of contract, difficulty in work resumption and supply chain interruptions.

According to the characteristics of small and medium-sized construction enterprises, this study establishes the growth evaluation index system, and constructs the growth evaluation model based on factor analysis. Through an analysis of the quarterly growth of small and medium-sized construction companies, this paper studies the impact of COVID-19 on the growth of enterprises and concludes that the epidemic had a greater impact on the growth of construction companies in the short term. In the long run, the impact of the epidemic on the growth of small and medium-sized construction enterprises has been limited. The epidemic has little impact on the solvency, tangible resources and intangible resources of small and medium-sized construction enterprises. The short-term negative impact of the epidemic on the profitability, capital expansion and market expectation of small and medium-sized construction enterprises is large, but the long-term impact is small. At the same time, overall, COVID-19 is conducive to the improvement of enterprises’ operating capacity. This study also has some limitations. For example, the research object only choose the small and medium-sized construction enterprises. Different industries have different characteristics, and influence of the COVID-19 is also different. In addition, the research object is Chinese enterprises, and overseas enterprises should be considered in the future. Despite these limitations, this study provides a reference for the growth impact of similar macro emergencies on small and medium-sized construction enterprises and the research foundation enriches the research content of the growth of small and medium-sized construction enterprises to a certain extent. The research results will help enterprise stakeholders understand the impact of the COVID-19 on the growth of construction enterprises, and provide decision support for the healthy and orderly development of the follow-up construction industry.

### 5.2 Countermeasures and suggestions

The impact of the epidemic on industrial development presents industry heterogeneity. Generally speaking, traditional manufacturing and service industries that require producers or consumers to be on-site are more vulnerable to the epidemic [56], such as construction, tourism, accommodation and catering industry, etc. According to the characteristics of the construction industry, the adverse effects of COVID-19 on small and medium-sized construction enterprises mainly include slow progress in construction, increasing costs, difficulty in resuming work, difficulty in labor service, difficulty in capital turnover, difficulty in supply chain protection, and difficulty in procurement of epidemic prevention materials [58]. the government and enterprises should make comprehensive use of policies and actively respond to negative effects. Therefore, combined with the conclusions of this study, the following suggestions are put forward:

Policy level: (1) continue to strengthen the epidemic prevention and control at the work site of construction projects, ensure stable and controllable epidemic prevention measures and production safety, and ensure that the relevant policies and measures to strengthen the epidemic prevention and control are implemented in enterprises and projects to prevent a rebound in the epidemic. (2) Take prevention and control as the premise, make full use of the Internet, simplify processing, compress approval times, and realize one-stop service. (3) Introduce corresponding policies to clarify the starting and ending points of the extended construction period and calculate the shutdown losses so that enterprises can reduce losses and disputes while ensuring the smooth implementation of existing construction contracts. (4) Strengthen informatization in the construction industry, promote the construction of an industry supply chain platform, and establish a centralized procurement and financial service platform for the construction industry. Integrate anti-epidemic materials into the supply chain to ensure adequate epidemic prevention materials. (5) Strengthen support for small and medium-sized construction enterprises and optimize the business environment for engineering construction, especially the development environment of small and medium-sized construction enterprises. During the epidemic period, while small and medium-sized enterprises are experiencing temporary business difficulties, the government should continue lending, not withdraw loans, improve the loan balance of financial institutions for small and medium-sized enterprises, provide long-term support policies such as interest free loans or extended repayment, actively adjust tax policy, appropriately reduce legal taxes for small and medium-sized construction enterprises, and offer employment subsidies.

At the enterprise level, small and medium-sized construction enterprises (1) should examine the problems that have accumulated during their development, actively adjust their layout, and grasp the development opportunities that emerge after the epidemic; (2) raise funds in various ways to ensure enterprise operations and the resumption of work and production, make full preparations for construction and epidemic prevention work, and strengthen quarterly follow-up measures to make up for construction delays; (3) make use of policy dividends to effectively compensate for the losses caused by the epidemic, take advantage of the policies issued by government departments to address the adverse effects of the epidemic, apply for support, and assign special personnel to fully study and interpret the relevant government policies to ensure they are being fully accessed; and (4) appropriately strengthen staff mental health management and assistance, provide appropriate material and spiritual support to employees and families affected by the epidemic, and minimize the negative emotions caused by the epidemic.

In short, the epidemic is not over, and globally, the situation is still grim. Construction enterprises should dynamically adjust their strategies of prevention and control, production and operation based on the epidemic situation. Enterprises also need to make full use of big data, intelligence and information technology to improve project management and controls, emergency response and risk management during public health and safety emergencies to realize the transformation and upgrading of construction enterprises.

## Supporting information

S1 Data (DOCX)
