# Supplementary material for: Study on the influence of COVID-19 on the growth of China’s small and medium-sized construction enterprises
Source: PLoS One. 2022 Jun 3;17(6):e0266315. doi: 10.1371/journal.pone.0266315 (PMC9165814; doi:10.1371/journal.pone.0266315)
Supplement: S1 Data — (DOCX) [file pone.0266315.s001.docx]

**The datasets of our present study**

Tables 1 to 7 are Original data for each quarter.

| Table 1 Original data of indicators in the first quarter of 2019 | | | | | | | | | | |
| --- | --- | --- | --- | --- | --- | --- | --- | --- | --- | --- |
| Enterprises | The ratio of intangible assets | The growth rate of intangible assets | Increasing rate of fixed assets | Cash ratio | Inventory | Accounts receivable | Number of employees | Current asset turnover | Turnover of fixed assets | Inventory turnover |
| BAUING | 2.1937 | -6.30% | -2.10% | 0.4268 | 379369179.9200 | 6366532823.2500 | 2089 | 0.1610 | 26.7923 | 3.3949 |
| CAMC | 2.8062 | -8.13% | -0.64% | 0.7758 | 2841085598.2300 | 4758547357.7200 | 2299 | 0.1476 | 1.5013 | 0.6335 |
| GHE | 1.4754 | -0.27% | -0.94% | 0.1085 | 3126798647.2600 | 2164704362.2300 | 4047 | 0.3205 | 0.3486 | 0.8834 |
| ZCT | 1.8515 | -1.35% | -0.86% | 0.2339 | 8237242546.3600 | 3754686541.1100 | 6597 | 0.2313 | 1.3420 | 0.5096 |
| HR | 0.2050 | -0.69% | 0.84% | 0.2489 | 7463021172.7900 | 605689633.4000 | 2157 | 0.2291 | 2.0857 | 0.3098 |
| Gold Mantis | 1.7253 | -2.59% | -1.25% | 0.3477 | 180263985.5100 | 18548576159.5100 | 19877 | 0.2139 | 7.0781 | 27.2311 |
| China Haisum | 1.2174 | -2.32% | -1.67% | 0.4378 | 508551916.4600 | 706092009.9200 | 4784 | 0.2399 | 4.1926 | 1.8564 |
| ZSSF | 2.0052 | -1.14% | -2.00% | 0.2768 | 3174791465.0600 | 2505463143.3500 | 5110 | 0.2331 | 1.0457 | 0.5018 |
| DHC | 2.4895 | -0.68% | -1.72% | 0.3787 | 1760824009.4300 | 647942945.2800 | 1167 | 0.0959 | 2.3316 | 0.2176 |
| Yanhua Smartech | 14.4802 | -4.46% | -12.79% | 0.4626 | 476133965.6000 | 401914751.2500 | 880 | 0.1271 | 1.6478 | 0.3773 |
| BXLQ | 0.5348 | 21.14% | -2.82% | 0.3440 | 3794946634.7000 | 2747865953.2800 | 4850 | 0.1177 | 2.2357 | 0.3664 |
| Orient Landscape | 7.4444 | 4.52% | -5.57% | 0.0704 | 14930777686.3800 | 8046045732.5200 | 5244 | 0.0387 | 0.5915 | 0.0484 |
| HONGTAO | 7.7552 | -0.84% | -1.49% | 0.1576 | 50960581.4300 | 5795329805.6000 | 2340 | 0.1406 | 0.8640 | 25.0550 |
| YASHA | 2.9203 | -1.08% | -1.25% | 0.1961 | 1979109038.5800 | 11453046779.5400 | 6153 | 0.1146 | 2.5485 | 0.9016 |
| PALM | 4.2817 | -0.30% | -3.11% | 0.1401 | 7095065019.8100 | 1644982802.8400 | 1557 | 0.0362 | 0.6806 | 0.0579 |
| Sanwei | 3.4853 | -4.15% | -0.98% | 1.0998 | 106535758.5300 | 439452280.1000 | 464 | 0.0829 | 0.9139 | 0.8366 |
| GRANDLAND | 6.0659 | -1.06% | -3.30% | 0.3580 | 1194860577.5700 | 9498525229.2000 | 5624 | 0.1280 | 29.0728 | 1.6791 |
| HOLU | 6.3210 | -0.50% | -0.73% | 0.2208 | 3828018773.8800 | 1727004560.2300 | 11053 | 0.2980 | 0.9724 | 0.5071 |
| ZHCGE | 8.9013 | -1.28% | -3.93% | 0.2534 | 2044253551.6100 | 2059900464.2400 | 2574 | 0.1373 | 0.6649 | 0.3184 |
| QDDFTT | 38.6324 | -0.26% | -3.13% | 0.4766 | 1224613414.3500 | 706865455.3300 | 2012 | 0.1289 | 0.1635 | 0.2471 |
| Ruihe | 1.3777 | -1.26% | -1.34% | 0.4500 | 255669221.6700 | 2401233010.7800 | 676 | 0.2145 | 1.0678 | 2.9145 |
| CDR&B | 0.0355 | -0.95% | -2.05% | 0.5544 | 867954015.4800 | 603565201.4900 | 626 | 0.1194 | 9.3850 | 0.4527 |
| Pbland | 16.5003 | 1.93% | -0.77% | 0.5893 | 2533147353.4000 | 1584263879.3600 | 2397 | 0.0872 | 1.5629 | 0.1828 |

| Continued Table 1 Original data of indicators in the first quarter of 2019 | | | | | | | | | | |
| --- | --- | --- | --- | --- | --- | --- | --- | --- | --- | --- |
| Enterprises | Accounts receivable turnover | Operating gross profit margin | Return on equity | Net profit margin on sales | Return on assets | Price/book value ratio (PB) | Price earning (PE) ratio | Asset-liability ratio | Current ratio | Quick ratio |
| BAUING | 0.2380 | 17.2356 | 2.1411 | 5.7527 | 1.6348 | 2.3795 | 27.9078 | 59.0476 | 1.8209 | 1.7483 |
| CAMC | 0.5081 | 20.6845 | 2.4448 | 9.6631 | 1.8369 | 1.8125 | 18.7512 | 49.4763 | 1.7567 | 1.4398 |
| GHE | 1.3392 | 8.1295 | 1.0114 | 1.1831 | 0.7259 | 1.3724 | 34.1402 | 84.6680 | 0.8284 | 0.5425 |
| ZCT | 1.3633 | 6.6957 | 0.7141 | 0.9918 | 0.5591 | 1.6754 | 58.8630 | 72.4765 | 1.0609 | 0.6230 |
| HR | 3.0291 | 9.3210 | 3.1131 | 3.7112 | 0.9295 | 1.5859 | 12.9362 | 78.5037 | 1.1257 | 0.3602 |
| Gold Mantis | 0.3260 | 18.2698 | 4.4396 | 9.1248 | 2.0463 | 2.2774 | 13.1137 | 55.5345 | 1.5807 | 1.5704 |
| China Haisum | 1.2780 | 12.0682 | 3.2950 | 4.9691 | 1.3389 | 3.1359 | 24.2292 | 66.7742 | 1.3894 | 1.2179 |
| ZSSF | 0.6862 | 10.6838 | 2.1430 | 4.7987 | 1.0998 | 1.5713 | 18.5270 | 59.7212 | 1.2607 | 0.7436 |
| DHC | 0.6582 | 17.7512 | 1.4478 | 7.2012 | 0.6117 | 1.7177 | 29.8739 | 64.7976 | 1.3955 | 0.8554 |
| Yanhua Smartech | 0.4754 | 5.9284 | -4.1999 | -23.6609 | -2.2578 | 4.1652 | -24.2726 | 46.5747 | 1.7622 | 1.1892 |
| BXLQ | 0.5729 | 11.9502 | 0.3399 | 1.3231 | 0.3412 | 3.9579 | 291.4430 | 84.5997 | 1.0476 | 0.7438 |
| Orient Landscape | 0.1191 | 28.5380 | -2.1318 | -26.8078 | -0.1158 | 1.7055 | -19.7880 | 69.2867 | 1.0272 | 0.4276 |
| HONGTAO | 0.2091 | 24.3307 | 2.1616 | 6.8948 | 1.2837 | 1.4776 | 16.6486 | 69.0412 | 1.2723 | 1.2648 |
| YASHA | 0.1670 | 14.7327 | 1.0668 | 4.2100 | 0.5859 | 1.1554 | 26.8491 | 59.0650 | 1.4439 | 1.2707 |
| PALM | 0.2335 | 8.1236 | -3.0364 | -38.9803 | -0.5577 | 1.3011 | -10.5428 | 67.5356 | 1.2921 | 0.5332 |
| Sanwei | 0.2859 | 27.0403 | 0.4954 | 3.4837 | 0.2374 | 2.0069 | 101.5293 | 21.8217 | 3.9804 | 3.6909 |
| GRANDLAND | 0.2252 | 15.3155 | 0.7543 | 2.3485 | 0.7428 | 1.3979 | 46.3435 | 64.0229 | 1.5804 | 1.4673 |
| HOLU | 1.2779 | 10.6642 | 1.5230 | 3.1821 | 1.0264 | 1.0646 | 17.6088 | 59.7565 | 1.2639 | 0.6170 |
| ZHCGE | 0.3496 | 18.4711 | 1.0564 | 5.2946 | 1.0337 | 2.7916 | 63.7042 | 52.0852 | 1.5388 | 0.9650 |
| QDDFTT | 1.0705 | 29.6603 | 0.2979 | 5.4857 | 0.3606 | 1.2528 | 106.7488 | 35.3869 | 1.3744 | 0.8501 |
| Ruihe | 0.3683 | 14.2410 | 2.1304 | 5.8532 | 1.3279 | 1.1725 | 13.9350 | 53.3463 | 1.4455 | 1.3544 |
| CDR&B | 0.7721 | 3.6866 | 0.3350 | 1.9067 | 0.3678 | 1.6225 | 121.3600 | 55.9165 | 1.5707 | 1.2207 |
| Pbland | 0.3029 | 11.0057 | 0.1816 | 2.1178 | 0.3396 | 1.3040 | 179.5916 | 39.2701 | 2.4983 | 1.4179 |

| Table 2 Original data of indicators in the second quarter of 2019 | | | | | | | | | | |
| --- | --- | --- | --- | --- | --- | --- | --- | --- | --- | --- |
| Enterprises | The ratio of intangible assets | The growth rate of intangible assets | Increasing rate of fixed assets | Cash ratio | Inventory | Accounts receivable | Number of employees | Current asset turnover | Turnover of fixed assets | Inventory turnover |
| BAUING | 1.2663 | 56.31% | -1.58% | 0.3560 | 370459739.1600 | 7164494900.3400 | 2089 | 0.3436 | 59.7120 | 7.5570 |
| CAMC | 2.8884 | 12.77% | 8.78% | 0.6456 | 3838460439.0400 | 5797785850.0800 | 2299 | 0.3266 | 3.5015 | 1.3226 |
| GHE | 1.4126 | -1.54% | -1.33% | 0.1786 | 3106811105.2800 | 2545437188.4400 | 4047 | 0.5357 | 0.6302 | 1.5546 |
| ZCT | 2.3148 | -1.42% | -1.66% | 0.2027 | 9690418491.5400 | 3913263937.0000 | 6597 | 0.5233 | 3.1568 | 1.0904 |
| HR | 0.2012 | -0.70% | 6.15% | 0.2214 | 7871536513.9100 | 645689633.4000 | 2157 | 0.3935 | 3.5316 | 0.5217 |
| Gold Mantis | 1.6598 | -2.71% | -1.35% | 0.3803 | 165695850.5000 | 20347099448.7500 | 19877 | 0.4695 | 16.2301 | 64.0932 |
| China Haisum | 1.1735 | -2.00% | -1.15% | 0.4059 | 576320545.2300 | 813903146.3700 | 4784 | 0.5941 | 10.3661 | 4.2901 |
| ZSSF | 1.9820 | -1.21% | -2.48% | 0.2322 | 3642504599.8900 | 2846565636.9200 | 5110 | 0.5055 | 2.3942 | 1.0382 |
| DHC | 2.3870 | 0.01% | -1.81% | 0.4648 | 1610820390.1500 | 618899599.9000 | 1167 | 0.2947 | 7.6088 | 0.7610 |
| Yanhua Smartech | 4.9089 | -6.15% | -3.78% | 0.4083 | 474338548.7500 | 432778050.0700 | 880 | 0.2652 | 3.4558 | 0.6939 |
| BXLQ | 0.4895 | 36.40% | 0.65% | 0.2890 | 3917573702.8800 | 2947865953.2800 | 4850 | 0.2888 | 5.4236 | 0.8826 |
| Orient Landscape | 6.2098 | 7.52% | -40.40% | 0.0591 | 14383283932.9600 | 8988488801.5300 | 5244 | 0.0825 | 1.5907 | 0.1058 |
| HONGTAO | 5.7884 | 22.11% | -0.54% | 0.1853 | 29815153.2500 | 5826656641.5200 | 2340 | 0.2220 | 1.3840 | 58.0264 |
| YASHA | 3.0121 | -6.40% | -0.66% | 0.2255 | 2028801302.9200 | 12010345129.9500 | 6153 | 0.2653 | 6.1383 | 2.1504 |
| PALM | 0.8293 | -1.15% | -1.87% | 0.1172 | 7631309998.1700 | 1592282637.7100 | 1557 | 0.1065 | 2.0413 | 0.1557 |
| Sanwei | 3.3997 | -1.56% | -1.45% | 1.1154 | 145889676.2200 | 398881536.7000 | 464 | 0.2142 | 2.3165 | 1.8353 |
| GRANDLAND | 4.7887 | -1.02% | -2.56% | 0.3338 | 981706698.1600 | 10326934812.1900 | 5624 | 0.3336 | 76.5302 | 4.9791 |
| HOLU | 5.8725 | 15.56% | 5.38% | 0.2160 | 4120022763.2800 | 1677377762.8400 | 11053 | 0.6543 | 2.1214 | 1.0733 |
| ZHCGE | 8.7145 | -0.98% | 4.69% | 0.2335 | 2018037551.5100 | 2470061888.9600 | 2574 | 0.3055 | 1.4863 | 0.7299 |
| QDDFTT | 35.3197 | -0.17% | -0.05% | 0.5217 | 989488549.4300 | 726865455.3300 | 2012 | 0.4587 | 0.5922 | 0.9763 |
| Ruihe | 0.9192 | -2.03% | -0.88% | 0.4730 | 265258104.6800 | 2389815175.3800 | 676 | 0.4325 | 2.1903 | 5.7317 |
| CDR&B | 0.0338 | -0.96% | -4.85% | 0.4909 | 1034329818.6200 | 481339721.9000 | 626 | 0.3132 | 25.4704 | 1.0954 |
| Pbland | 17.8434 | 9.24% | -10.24% | 0.5202 | 2667352691.1900 | 1680305297.5400 | 2397 | 0.2200 | 4.1634 | 0.4217 |

| Continued Table 2 Original data of indicators in the second quarter of 2019 | | | | | | | | | | |
| --- | --- | --- | --- | --- | --- | --- | --- | --- | --- | --- |
| Enterprises | Accounts receivable turnover | Operating gross profit margin | Return on equity | Net profit margin on sales | Return on assets | Price/book value ratio (PB) | Price earning (PE) ratio | Asset-liability ratio | Current ratio | Quick ratio |
| BAUING | 0.4946 | 17.7141 | 4.9161 | 6.0062 | 3.3506 | 1.8454 | 19.0546 | 60.8700 | 1.7502 | 1.6865 |
| CAMC | 1.1075 | 19.9857 | 5.2191 | 8.9855 | 2.6155 | 1.4060 | 14.2051 | 53.4688 | 1.6532 | 1.3159 |
| GHE | 2.2046 | 10.2758 | 2.7266 | 1.7613 | 1.5908 | 1.2552 | 23.1655 | 85.6554 | 0.8950 | 0.6267 |
| ZCT | 3.1121 | 7.5092 | 3.2681 | 2.0539 | 1.5853 | 1.1435 | 17.5789 | 72.2175 | 1.0805 | 0.5885 |
| HR | 7.0793 | 10.0677 | 5.0955 | 3.3972 | 1.4533 | 1.4002 | 13.8500 | 79.5076 | 1.0964 | 0.3348 |
| Gold Mantis | 0.7083 | 18.9247 | 8.1789 | 7.8522 | 3.9614 | 1.9920 | 12.4561 | 58.2141 | 1.5432 | 1.5346 |
| China Haisum | 2.9332 | 11.2324 | 6.8980 | 4.1435 | 2.6032 | 3.0320 | 21.9228 | 67.7484 | 1.3673 | 1.1733 |
| ZSSF | 1.4581 | 12.3865 | 4.6610 | 4.6641 | 2.4131 | 1.3618 | 14.9173 | 61.2749 | 1.2659 | 0.7175 |
| DHC | 2.1772 | 15.1761 | 4.1341 | 6.3839 | 1.6658 | 2.1682 | 26.4926 | 67.1320 | 1.3470 | 0.9145 |
| Yanhua Smartech | 0.9439 | 16.1611 | -2.4468 | -6.5394 | -1.1848 | 3.3865 | -68.3078 | 45.2170 | 1.8508 | 1.2352 |
| BXLQ | 1.1459 | 11.2953 | 1.1380 | 0.4893 | 0.5805 | 3.3384 | 142.0975 | 86.0569 | 1.0478 | 0.7297 |
| Orient Landscape | 0.2439 | 29.0464 | -7.3412 | -40.8805 | -1.0275 | 1.3890 | -9.0115 | 70.9331 | 1.0425 | 0.4723 |
| HONGTAO | 0.3332 | 22.4881 | 2.4563 | 5.1192 | 1.7911 | 1.2281 | 24.5349 | 68.6221 | 1.3481 | 1.3435 |
| YASHA | 0.3918 | 14.1622 | 2.3789 | 4.0514 | 1.3132 | 1.0145 | 21.2238 | 61.3836 | 1.3994 | 1.2396 |
| PALM | 0.7037 | 13.7347 | -3.4841 | -15.4862 | -0.4616 | 1.0991 | -15.4831 | 68.5760 | 1.2568 | 0.4792 |
| Sanwei | 0.7556 | 24.5979 | 1.8231 | 7.2014 | 1.4303 | 1.8261 | 49.4789 | 20.5902 | 4.1792 | 3.7402 |
| GRANDLAND | 0.5615 | 12.6818 | 2.5051 | 3.1418 | 1.5604 | 1.0626 | 21.3386 | 63.9784 | 1.4683 | 1.3814 |
| HOLU | 2.9059 | 12.3037 | 3.7811 | 3.5493 | 2.3404 | 0.9108 | 12.2115 | 61.8815 | 1.1872 | 0.5577 |
| ZHCGE | 0.7312 | 18.8273 | 2.6193 | 5.7471 | 2.1721 | 2.1556 | 39.8576 | 53.0435 | 1.5211 | 0.9910 |
| QDDFTT | 2.1405 | 31.3436 | 3.0172 | 15.9233 | 2.6140 | 1.0501 | 17.6226 | 35.4672 | 1.3760 | 0.9658 |
| Ruihe | 0.7541 | 15.8530 | 4.2895 | 6.1314 | 2.7903 | 1.0534 | 12.4411 | 53.9514 | 1.4391 | 1.3474 |
| CDR&B | 2.2745 | 4.6584 | 0.9275 | 2.0115 | 0.8787 | 1.4672 | 79.5904 | 58.0047 | 1.3997 | 1.0354 |
| Pbland | 0.7451 | 16.6400 | 1.7314 | 6.8645 | 1.5979 | 1.0461 | 30.4541 | 38.6438 | 2.5442 | 1.3906 |

| Table 3 Original data of indicators in the third quarter of 2019 | | | | | | | | | | |
| --- | --- | --- | --- | --- | --- | --- | --- | --- | --- | --- |
| Enterprises | The ratio of intangible assets | The growth rate of intangible assets | Increasing rate of fixed assets | Cash ratio | Inventory | Accounts receivable | Number of employees | Current asset turnover | Turnover of fixed assets | Inventory turnover |
| BAUING | 1.2093 | -12.54% | -1.84% | 0.3656 | 416663342.3900 | 7039787869.6700 | 2089 | 0.5176 | 90.0486 | 10.6463 |
| CAMC | 2.6749 | -3.62% | -0.39% | 0.6696 | 4052892211.5600 | 5431991674.4300 | 2299 | 0.4516 | 4.9067 | 1.8001 |
| GHE | 1.2975 | -1.03% | 8.54% | 0.1621 | 3151575025.4800 | 2679434030.7000 | 4047 | 0.8298 | 0.9335 | 2.3816 |
| ZCT | 2.1817 | -0.94% | 0.39% | 0.2139 | 10402809495.9800 | 4330232752.2600 | 6597 | 0.8312 | 5.2372 | 1.7463 |
| HR | 0.1940 | -0.88% | 2.42% | 0.2272 | 7582080359.3200 | 615672014.8100 | 2157 | 0.6542 | 5.6960 | 0.8425 |
| Gold Mantis | 1.5735 | 4.06% | -1.33% | 0.3415 | 133895241.7300 | 22690979713.7200 | 19877 | 0.7480 | 26.9307 | 116.7381 |
| China Haisum | 1.1638 | -1.04% | -0.66% | 0.4477 | 576821920.0900 | 804025818.3400 | 4784 | 0.8312 | 15.1003 | 6.1506 |
| ZSSF | 1.8427 | -1.17% | -2.36% | 0.1968 | 3988621914.3900 | 2467147542.9500 | 5110 | 0.7972 | 3.7631 | 1.5511 |
| DHC | 2.2151 | -1.57% | -0.96% | 0.4222 | 1622429043.5000 | 743027573.1300 | 1167 | 0.4996 | 13.0293 | 1.3397 |
| Yanhua Smartech | 5.1267 | -17.33% | 0.18% | 0.4255 | 493027081.6100 | 443592095.0300 | 880 | 0.4011 | 5.3273 | 1.0305 |
| BXLQ | 0.6421 | -2.98% | -0.95% | 0.2762 | 4118630296.0500 | 3249858023.4300 | 4850 | 0.5439 | 10.4253 | 1.6724 |
| Orient Landscape | 5.3883 | 15.50% | 20.91% | 0.0525 | 14574448641.9800 | 9306467502.6500 | 5244 | 0.1450 | 2.5902 | 0.1866 |
| HONGTAO | 5.7670 | -1.14% | -1.29% | 0.2063 | 34856023.8200 | 5904508146.0000 | 2340 | 0.3379 | 2.1268 | 82.7235 |
| YASHA | 2.7442 | -0.86% | -1.36% | 0.1863 | 2129990832.2800 | 12280960361.7500 | 6153 | 0.4222 | 9.8240 | 3.3224 |
| PALM | 0.8015 | -17.24% | 0.79% | 0.1011 | 7538588409.6400 | 1493899273.8300 | 1557 | 0.1671 | 3.1508 | 0.2404 |
| Sanwei | 3.5200 | -3.26% | -0.27% | 1.3616 | 113554460.5500 | 411758933.0200 | 464 | 0.3242 | 3.4979 | 3.0517 |
| GRANDLAND | 4.7391 | -1.04% | -0.24% | 0.3149 | 1059865595.4900 | 10948386103.6100 | 5624 | 0.5202 | 121.7856 | 7.5826 |
| HOLU | 6.3472 | -0.49% | 2.99% | 0.1714 | 4364875011.3400 | 1657515987.7200 | 11053 | 1.0444 | 3.3408 | 1.6456 |
| ZHCGE | 8.2378 | -1.31% | -1.09% | 0.2344 | 2147314362.8500 | 2435423833.2100 | 2574 | 0.4477 | 2.2085 | 1.0287 |
| QDDFTT | 35.4105 | -0.20% | 0.25% | 0.4142 | 1313509433.8300 | 566383106.9500 | 2012 | 0.5802 | 0.7217 | 1.0039 |
| Ruihe | 0.8970 | -1.69% | -1.48% | 0.4648 | 274917400.4800 | 2568666724.6100 | 676 | 0.6436 | 3.3675 | 8.5869 |
| CDR&B | 0.0315 | 2.51% | -3.11% | 0.3969 | 977100356.9100 | 461387130.9600 | 626 | 0.5001 | 40.1166 | 1.7349 |
| Pbland | 18.3181 | 14.95% | -10.46% | 0.5993 | 2637432448.1600 | 1653733983.6500 | 2397 | 0.3432 | 6.9902 | 0.6867 |

| Continued Table 3 Original data of indicators in the third quarter of 2019 | | | | | | | | | | |
| --- | --- | --- | --- | --- | --- | --- | --- | --- | --- | --- |
| Enterprises | Accounts receivable turnover | Operating gross profit margin | Return on equity | Net profit margin on sales | Return on assets | Price/book value ratio (PB) | Price earning (PE) ratio | Asset-liability ratio | Current ratio | Quick ratio |
| BAUING | 0.7462 | 17.4047 | 6.9879 | 5.7527 | 5.1770 | 1.6408 | 18.0909 | 59.3599 | 1.8012 | 1.7264 |
| CAMC | 1.6081 | 19.6198 | 7.7931 | 9.7271 | 2.6939 | 1.1885 | 12.2100 | 53.0736 | 1.6679 | 1.3158 |
| GHE | 3.3066 | 10.2732 | 3.8218 | 1.6385 | 2.4954 | 1.1851 | 23.5948 | 85.8889 | 0.8774 | 0.6090 |
| ZCT | 4.8961 | 7.6276 | 6.4095 | 2.5537 | 2.7709 | 0.9614 | 11.4826 | 73.6160 | 1.0830 | 0.5999 |
| HR | 11.8493 | 12.7815 | 7.6223 | 3.7688 | 2.9291 | 1.2055 | 12.1095 | 78.3873 | 1.0974 | 0.3367 |
| Gold Mantis | 1.1012 | 18.5789 | 12.6103 | 7.6504 | 6.1609 | 1.6711 | 10.4020 | 58.9693 | 1.5371 | 1.5306 |
| China Haisum | 4.2769 | 12.1429 | 11.0994 | 4.7057 | 3.9360 | 2.2015 | 15.1729 | 68.3053 | 1.3627 | 1.1819 |
| ZSSF | 2.4287 | 11.5521 | 5.6689 | 3.6691 | 3.0649 | 1.3359 | 18.1378 | 60.0697 | 1.3157 | 0.6731 |
| DHC | 3.3803 | 12.0514 | 5.9630 | 5.4535 | 2.2529 | 1.8667 | 23.9369 | 66.8802 | 1.3507 | 0.9182 |
| Yanhua Smartech | 1.4383 | 17.7717 | -1.8616 | -3.0967 | -0.8082 | 3.0732 | -122.5759 | 46.5273 | 1.8062 | 1.2073 |
| BXLQ | 2.0887 | 9.6247 | 1.9791 | 0.4636 | 0.9104 | 2.9045 | 107.6872 | 85.7780 | 0.9790 | 0.6755 |
| Orient Landscape | 0.4196 | 28.0974 | -7.2614 | -23.2571 | -0.4946 | 1.1703 | -11.5318 | 71.2953 | 1.0525 | 0.4650 |
| HONGTAO | 0.5053 | 20.4659 | 3.3224 | 4.6739 | 2.5336 | 1.1966 | 26.6441 | 68.6072 | 1.3702 | 1.3648 |
| YASHA | 0.6159 | 14.3340 | 3.7712 | 4.1241 | 2.0510 | 0.9089 | 18.1190 | 60.7171 | 1.4163 | 1.2462 |
| PALM | 1.1198 | 14.5741 | -3.3941 | -10.0762 | -0.0178 | 0.8630 | -18.7451 | 68.0892 | 1.2241 | 0.4570 |
| Sanwei | 1.1216 | 27.5482 | 3.3155 | 8.8062 | 2.7286 | 1.6380 | 36.8894 | 18.7841 | 4.5823 | 4.2047 |
| GRANDLAND | 0.8660 | 13.0740 | 4.5901 | 3.4946 | 3.0708 | 0.9935 | 16.5023 | 64.4898 | 1.4579 | 1.3684 |
| HOLU | 4.6744 | 13.2718 | 7.3971 | 4.4244 | 4.3699 | 0.8949 | 9.3647 | 60.8800 | 1.2061 | 0.5307 |
| ZHCGE | 1.0884 | 20.0239 | 4.2560 | 6.4075 | 3.4443 | 1.8053 | 31.1125 | 52.7939 | 1.5283 | 0.9710 |
| QDDFTT | 2.9704 | 32.7695 | 3.4006 | 14.7109 | 2.9762 | 1.0476 | 23.3892 | 34.2772 | 1.4076 | 0.8113 |
| Ruihe | 1.1083 | 15.8530 | 6.2856 | 6.1159 | 4.1421 | 0.9531 | 11.6567 | 54.4065 | 1.4455 | 1.3546 |
| CDR&B | 3.5945 | 5.2532 | 1.1835 | 1.6527 | 1.3897 | 1.2843 | 82.1251 | 57.9315 | 1.3029 | 0.9634 |
| Pbland | 1.1982 | 15.4473 | 2.0096 | 5.0223 | 1.9760 | 0.9940 | 37.4657 | 40.6932 | 2.3866 | 1.3650 |

| Table 4 Original data of indicators in the fourth quarter of 2019 | | | | | | | | | | |
| --- | --- | --- | --- | --- | --- | --- | --- | --- | --- | --- |
| Enterprises | The ratio of intangible assets | The growth rate of intangible assets | Increasing rate of fixed assets | Cash ratio | Inventory | Accounts receivable | Number of employees | Current asset turnover | Turnover of fixed assets | Inventory turnover |
| BAUING | 1.2258 | -11.94% | -2.71% | 0.3620 | 377825568.7900 | 6796473313.1500 | 2071 | 0.7117 | 124.4368 | 15.4857 |
| CAMC | 2.5943 | 15.11% | -0.03% | 0.7391 | 4205958132.8300 | 4865762237.5900 | 5761 | 0.6410 | 6.7997 | 2.3615 |
| GHE | 1.2419 | -1.74% | 7.00% | 0.1913 | 3220445935.3800 | 3347004776.9800 | 4220 | 1.0804 | 1.2897 | 3.3430 |
| ZCT | 1.9918 | -2.06% | 1.73% | 0.2269 | 11085392253.1900 | 4516144473.9800 | 6968 | 1.1846 | 7.8657 | 2.5500 |
| HR | 0.1953 | -0.54% | -4.91% | 0.2761 | 7442247728.5100 | 1224938040.5000 | 2163 | 1.0390 | 9.7798 | 1.4480 |
| Gold Mantis | 1.5204 | -0.18% | 9.96% | 0.5225 | 101344129.2800 | 22003095138.8400 | 17254 | 0.9604 | 34.8162 | 176.8021 |
| China Haisum | 1.0822 | 2.73% | -0.01% | 0.6658 | 457694408.6100 | 771879202.0900 | 4866 | 1.4184 | 24.4756 | 11.7868 |
| ZSSF | 1.8592 | 0.75% | -0.15% | 0.1926 | 3741973071.7500 | 3155632357.7500 | 5338 | 1.0913 | 5.2863 | 2.2528 |
| DHC | 2.1573 | -1.04% | -1.50% | 0.3877 | 1750966924.9200 | 956936710.2900 | 1228 | 0.9670 | 25.1708 | 2.4626 |
| Yanhua Smartech | 5.2219 | 669.53% | -7.39% | 0.4657 | 441365432.9400 | 446788852.8700 | 965 | 0.5916 | 8.1471 | 1.5931 |
| BXLQ | 0.5958 | 3552.82% | 70.16% | 0.2631 | 4091142219.1300 | 1929533917.8600 | 4243 | 0.9471 | 12.9156 | 2.7863 |
| Orient Landscape | 6.0304 | 1.41% | -0.32% | 0.0492 | 16010628883.6800 | 9650126978.6400 | 3388 | 0.2978 | 5.4988 | 0.3699 |
| HONGTAO | 5.6926 | 30.51% | 10.47% | 0.1981 | 22938344.8300 | 5898525845.3200 | 1927 | 0.4868 | 2.8010 | 147.0884 |
| YASHA | 2.7403 | 1.93% | 5.92% | 0.2849 | 1983831185.7300 | 11683635899.7200 | 6847 | 0.5955 | 13.7051 | 4.9557 |
| PALM | 0.7029 | 186.80% | 2.54% | 0.1292 | 6608650890.5600 | 1588085656.8800 | 1269 | 0.2259 | 4.1424 | 0.3773 |
| Sanwei | 3.4722 | -4.17% | 5.12% | 2.5155 | 98586156.5100 | 416533559.4500 | 448 | 0.4365 | 4.6662 | 4.5814 |
| GRANDLAND | 4.5611 | 2.41% | 81.34% | 0.4344 | 922307651.3700 | 12272492050.8900 | 3999 | 0.6681 | 125.5840 | 11.6612 |
| HOLU | 6.2506 | 1.68% | 23.64% | 0.1819 | 4657119443.9000 | 1677450352.3400 | 11113 | 1.4291 | 4.1887 | 2.2110 |
| ZHCGE | 8.1145 | -18.28% | 7.50% | 0.2643 | 2101192694.4700 | 2833107670.6200 | 2005 | 0.6792 | 3.3572 | 1.6248 |
| QDDFTT | 36.0280 | -0.26% | 10.80% | 0.5890 | 1220750166.4900 | 608217945.2000 | 1912 | 0.8194 | 1.0286 | 1.7042 |
| Ruihe | 0.8612 | 0.55% | -1.87% | 0.4911 | 273986421.5500 | 2641435084.2400 | 699 | 0.8910 | 4.8407 | 12.2063 |
| CDR&B | 0.0319 | -1.03% | 3.89% | 0.2666 | 1061349059.2200 | 462111371.4400 | 690 | 0.7415 | 57.2803 | 2.4515 |
| Pbland | 18.6053 | 8.93% | -1.93% | 0.4996 | 2267246954.6200 | 1616953138.1100 | 2397 | 0.5222 | 10.2593 | 1.1553 |

| Continued Table 4 Original data of indicators in the fourth quarter of 2019 | | | | | | | | | | |
| --- | --- | --- | --- | --- | --- | --- | --- | --- | --- | --- |
| Enterprises | Accounts receivable turnover | Operating gross profit margin | Return on equity | Net profit margin on sales | Return on assets | Price/book value ratio (PB) | Price earning (PE) ratio | Asset-liability ratio | Current ratio | Quick ratio |
| BAUING | 1.0368 | 16.4034 | 5.1938 | 2.9914 | 4.4033 | 1.8927 | 37.0471 | 59.4815 | 1.6265 | 1.5643 |
| CAMC | 2.3683 | 22.2021 | 10.6507 | 9.8245 | 5.6128 | 1.1201 | 11.4144 | 49.9280 | 1.7513 | 1.3506 |
| GHE | 4.1453 | 10.9780 | 7.4528 | 2.2039 | 3.2925 | 1.1261 | 15.5671 | 86.9274 | 0.8988 | 0.6609 |
| ZCT | 7.2435 | 7.9397 | 9.7298 | 2.6636 | 4.0823 | 1.0453 | 11.1775 | 74.8079 | 1.1029 | 0.6364 |
| HR | 13.1657 | 11.2393 | 11.2924 | 3.2464 | 4.2615 | 1.2009 | 11.0308 | 78.8431 | 1.1105 | 0.4276 |
| Gold Mantis | 1.5185 | 18.3880 | 16.5513 | 7.3317 | 7.7243 | 1.5575 | 10.0477 | 60.9864 | 1.5146 | 1.5103 |
| China Haisum | 7.0637 | 8.5630 | 4.1512 | 1.0501 | 1.6876 | 2.3449 | 55.6514 | 67.6747 | 1.3576 | 1.1991 |
| ZSSF | 3.0150 | 11.5813 | 6.4360 | 2.9530 | 3.3980 | 1.3177 | 21.0764 | 61.4909 | 1.2637 | 0.7122 |
| DHC | 5.6199 | 12.1912 | 8.1864 | 3.9765 | 3.0912 | 1.6933 | 21.2345 | 66.2781 | 1.3005 | 0.8432 |
| Yanhua Smartech | 2.1180 | 18.4726 | 2.7087 | 1.2435 | 0.9957 | 3.4555 | 129.2066 | 48.9299 | 1.5889 | 1.1191 |
| BXLQ | 4.3873 | 10.0000 | 2.8322 | 0.4338 | 1.5203 | 2.8600 | 99.0663 | 86.0592 | 1.0002 | 0.6656 |
| Orient Landscape | 0.8732 | 29.4961 | 0.4122 | 0.5424 | 1.9684 | 1.0848 | 259.7239 | 71.0368 | 1.0657 | 0.4523 |
| HONGTAO | 0.7001 | 19.6269 | 1.1562 | 1.9787 | 1.9802 | 1.3269 | 109.8289 | 68.4281 | 1.3807 | 1.3769 |
| YASHA | 0.9064 | 14.3555 | 5.3552 | 4.1005 | 2.9459 | 0.9693 | 18.2584 | 61.7735 | 1.3865 | 1.2367 |
| PALM | 1.4529 | 5.7620 | -19.3964 | -38.9132 | -5.0678 | 0.9912 | -4.6210 | 72.3633 | 1.0758 | 0.4658 |
| Sanwei | 1.5251 | 25.9057 | 6.3610 | 12.5708 | 5.1544 | 1.6779 | 26.6710 | 19.0786 | 4.5073 | 4.1949 |
| GRANDLAND | 1.1710 | 13.1585 | 2.0729 | 1.0164 | 1.6052 | 0.9708 | 46.7989 | 71.1313 | 1.4432 | 1.3811 |
| HOLU | 6.5395 | 14.2075 | 11.9451 | 5.1987 | 6.4131 | 1.1232 | 9.9195 | 61.2780 | 1.1716 | 0.5073 |
| ZHCGE | 1.5846 | 20.8135 | 6.3013 | 6.0257 | 4.6894 | 1.7103 | 26.8486 | 53.9615 | 1.5197 | 1.0166 |
| QDDFTT | 4.3047 | 27.0025 | 4.0179 | 11.5775 | 3.6999 | 1.2192 | 30.8229 | 34.6251 | 1.5142 | 0.9825 |
| Ruihe | 1.5551 | 16.1646 | 6.9336 | 4.8434 | 4.7826 | 0.9983 | 14.8121 | 55.6721 | 1.4340 | 1.3488 |
| CDR&B | 5.2236 | 4.2415 | 1.5851 | 1.5117 | 1.8526 | 1.2956 | 82.8140 | 59.4788 | 1.1318 | 0.7984 |
| Pbland | 1.7623 | 9.1730 | -21.7686 | -32.4288 | -11.3414 | 1.2747 | -5.2186 | 44.3995 | 1.9857 | 1.1852 |

| Table 5 Original data of indicators in the first quarter of 2020 | | | | | | | | | | |
| --- | --- | --- | --- | --- | --- | --- | --- | --- | --- | --- |
| Enterprises | The ratio of intangible assets | The growth rate of intangible assets | Increasing rate of fixed assets | Cash ratio | Inventory | Accounts receivable | Number of employees | Current asset turnover | Turnover of fixed assets | Inventory turnover |
| BAUING | 1.2125 | -10.92% | -3.16% | 0.4542 | 140709800.0600 | 5930453061.6800 | 2071 | 0.0947 | 18.3187 | 2.9778 |
| CAMC | 2.5943 | -5.20% | -4.18% | 0.6716 | 4444369478.5100 | 5217960118.7600 | 5761 | 0.0871 | 1.0084 | 0.2995 |
| GHE | 1.2419 | -1.13% | -1.47% | 0.1859 | 367303287.6400 | 3809229567.8500 | 4220 | 0.2586 | 0.3353 | 1.5554 |
| ZCT | 1.9918 | -1.38% | -2.47% | 0.2465 | 1264321218.2600 | 1400557580.8300 | 6968 | 0.2027 | 1.3169 | 0.7319 |
| HR | 0.1953 | -0.71% | -1.70% | 0.2829 | 3300038147.2700 | 1049125288.6700 | 2163 | 0.1966 | 1.9123 | 0.3970 |
| Gold Mantis | 1.5204 | -3.26% | -0.96% | 0.6129 | 101738647.3300 | 10833252467.0200 | 17254 | 0.1281 | 4.7702 | 35.5757 |
| China Haisum | 1.0822 | -2.84% | -1.93% | 0.6348 | 612964797.5100 | 694650358.0300 | 4866 | 0.1883 | 3.4730 | 1.2803 |
| ZSSF | 1.7598 | 31.67% | -2.13% | 0.1763 | 1738156208.1000 | 2802954604.2700 | 5338 | 0.2021 | 1.0942 | 0.5715 |
| DHC | 2.1411 | -0.06% | 0.13% | 0.3154 | 126858062.8900 | 936401064.9900 | 1228 | 0.0520 | 1.4656 | 0.1647 |
| Yanhua Smartech | 5.2219 | 1.21% | -5.51% | 0.4398 | 42408466.7600 | 417787569.3700 | 965 | 0.0887 | 1.3482 | 0.4876 |
| BXLQ | 0.5958 | -3.28% | -0.98% | 0.3123 | 2510896477.3800 | 1853840951.7700 | 4243 | 0.0709 | 0.8546 | 0.2329 |
| Orient Landscape | 6.0304 | 2.57% | 3.97% | 0.0694 | 502434821.8400 | 9251159762.2000 | 3388 | 0.0159 | 0.3620 | 0.0414 |
| HONGTAO | 5.6926 | -0.94% | -1.11% | 0.1553 | 29646895.8600 | 6019352724.8100 | 1927 | 0.0998 | 0.5580 | 24.8302 |
| YASHA | 2.7403 | -1.34% | -1.32% | 0.2409 | 1796953427.1900 | 3763822566.9000 | 6847 | 0.0840 | 1.8662 | 0.6610 |
| PALM | 0.7029 | 4.88% | 0.18% | 0.0833 | 1581793566.7300 | 1293851591.4800 | 1269 | 0.0252 | 0.4386 | 0.0733 |
| Sanwei | 3.4722 | -4.50% | -1.73% | 1.4677 | 50605485.7600 | 352084132.1000 | 448 | 0.0361 | 0.3715 | 0.3824 |
| GRANDLAND | 4.5611 | -1.24% | -1.82% | 0.3862 | 1103348543.6500 | 11847282662.9200 | 3999 | 0.0596 | 9.4853 | 1.0343 |
| HOLU | 6.2506 | -4.39% | -0.27% | 0.1482 | 4418759017.1300 | 1540947761.9800 | 11113 | 0.1693 | 0.4702 | 0.2665 |
| ZHCGE | 8.1145 | -1.53% | -0.10% | 0.2430 | 90578872.2300 | 2631307066.5800 | 2005 | 0.1003 | 0.5036 | 0.4575 |
| QDDFTT | 36.0280 | -0.19% | -0.47% | 0.6388 | 1241514567.1500 | 634834832.6000 | 1912 | 0.1396 | 0.2005 | 0.3131 |
| Ruihe | 0.8612 | -1.77% | -1.54% | 0.5717 | 263401461.9000 | 341213863.0100 | 699 | 0.0985 | 0.5709 | 1.3555 |
| CDR&B | 0.0319 | -1.04% | -5.08% | 0.2887 | 45526425.6500 | 434604331.3000 | 690 | 0.1084 | 7.7947 | 0.6172 |
| Pbland | 18.6053 | 0.16% | -1.93% | 0.5339 | 2257836772.8700 | 1305463222.8800 | 2397 | 0.0548 | 1.1303 | 0.1224 |

| Continued Table 5 Original data of indicators in the first quarter of 2020 | | | | | | | | | | |
| --- | --- | --- | --- | --- | --- | --- | --- | --- | --- | --- |
| Enterprises | Accounts receivable turnover | Operating gross profit margin | Return on equity | Net profit margin on sales | Return on assets | Price/book value ratio (PB) | Price earning (PE) ratio | Asset-liability ratio | Current ratio | Quick ratio |
| BAUING | 0.1457 | 16.7311 | 0.4225 | 1.7852 | 0.7030 | 1.5300 | 90.6815 | 58.7881 | 1.6374 | 1.6136 |
| CAMC | 0.3182 | 19.2621 | 0.2120 | 0.4773 | -0.2311 | 0.9535 | 112.2908 | 50.0950 | 1.7530 | 1.3309 |
| GHE | 0.8547 | 8.7655 | 1.1073 | 1.2223 | 0.7259 | 1.1758 | 26.7393 | 87.0422 | 0.8274 | 0.8009 |
| ZCT | 1.6117 | 5.2155 | 0.1281 | -0.0473 | 0.2448 | 0.9088 | 177.4959 | 69.1960 | 1.1857 | 1.1138 |
| HR | 2.0789 | 9.7959 | 2.9339 | 3.9635 | 0.8602 | 1.5799 | 13.6387 | 77.8433 | 1.1594 | 0.8391 |
| Gold Mantis | 0.2632 | 16.3966 | 2.1817 | 6.9417 | 1.0330 | 1.4295 | 16.5896 | 56.9382 | 1.6181 | 1.6130 |
| China Haisum | 1.0474 | 10.7581 | 1.8253 | 3.3382 | 0.6272 | 2.2265 | 30.7876 | 69.2252 | 1.3382 | 1.1446 |
| ZSSF | 0.5952 | 11.6895 | 2.3570 | 5.6917 | 1.1559 | 1.8963 | 20.3505 | 61.8491 | 1.2044 | 0.9711 |
| DHC | 0.2697 | 39.4077 | 1.4049 | 12.4604 | 0.5614 | 1.5393 | 27.8154 | 64.4256 | 1.3420 | 1.3069 |
| Yanhua Smartech | 0.2993 | 8.8216 | -2.2418 | -16.5839 | -0.9716 | 3.3736 | -37.1988 | 48.6570 | 1.5658 | 1.5193 |
| BXLQ | 0.4821 | 15.7039 | 0.2148 | 0.0322 | 0.2159 | 2.9161 | 340.0079 | 86.9758 | 1.0477 | 0.8530 |
| Orient Landscape | 0.0467 | 22.5435 | -2.3360 | -64.8235 | -0.2972 | 1.1940 | -12.6314 | 72.0214 | 1.1751 | 1.1538 |
| HONGTAO | 0.1384 | 20.8600 | 0.2039 | 1.8096 | 0.4942 | 1.2621 | 150.1551 | 68.7119 | 1.3722 | 1.3673 |
| YASHA | 0.1912 | 15.3965 | 0.6848 | 3.5329 | 0.3795 | 1.1678 | 42.7627 | 59.4755 | 1.3910 | 1.2423 |
| PALM | 0.1975 | -5.5595 | -5.0432 | -84.0774 | -0.8513 | 0.9009 | -4.3569 | 72.5292 | 1.0325 | 0.8831 |
| Sanwei | 0.1311 | 43.3754 | 0.3326 | 4.1784 | 0.1340 | 1.7172 | 129.2921 | 16.1653 | 5.3944 | 5.1951 |
| GRANDLAND | 0.1021 | 14.8820 | -1.3162 | -7.8400 | 0.1513 | 0.8356 | -15.7657 | 69.6438 | 1.5023 | 1.4190 |
| HOLU | 0.8559 | 12.1946 | 0.8376 | 3.0155 | 0.5788 | 1.6113 | 48.2925 | 60.6468 | 1.1759 | 0.5310 |
| ZHCGE | 0.2292 | 19.9487 | 0.9214 | 5.9547 | 0.8452 | 1.7144 | 44.9598 | 52.1224 | 1.5831 | 1.5598 |
| QDDFTT | 0.8449 | 26.5979 | 0.5906 | 8.5908 | 0.5486 | 1.2009 | 50.7419 | 37.3113 | 1.4382 | 0.9968 |
| Ruihe | 0.2912 | 16.1369 | 0.0821 | 1.4931 | 0.3174 | 0.9075 | 277.2142 | 51.9216 | 1.4881 | 1.3950 |
| CDR&B | 0.8004 | 4.8281 | 0.4526 | 4.2836 | 0.6530 | 1.1239 | 62.1758 | 56.9757 | 1.2128 | 1.1945 |
| Pbland | 0.2026 | 6.4881 | -0.8555 | -11.5659 | -0.2806 | 1.0650 | -30.9970 | 41.4733 | 2.2265 | 1.2575 |

| Table 6 Original data of indicators in the second quarter of 2020 | | | | | | | | | | |
| --- | --- | --- | --- | --- | --- | --- | --- | --- | --- | --- |
| Enterprises | The ratio of intangible assets | The growth rate of intangible assets | Increasing rate of fixed assets | Cash ratio | Inventory | Accounts receivable | Number of employees | Current asset turnover | Turnover of fixed assets | Inventory turnover |
| BAUING | 0.6713 | 324.1113643 | -0.0275323 | 0.4350 | 72,073,998.8500 | 3,406,750,446.8800 | 2,071.0000 | 0.2641 | 54.6715 | 10.1146 |
| CAMC | 2.7767 | 0.063135484 | 0.00243294 | 0.7297 | 4,153,518,652.0700 | 4,940,145,406.1100 | 5,761.0000 | 0.1957 | 2.2137 | 0.6900 |
| GHE | 1.0637 | -0.010851258 | -0.0073874 | 0.1482 | 765,263,921.5400 | 4,348,805,087.0900 | 4,220.0000 | 0.4784 | 0.6283 | 2.5360 |
| ZCT | 2.1687 | -0.014743455 | -0.0443301 | 0.3290 | 1,196,942,704.7100 | 1,791,718,067.1700 | 6,968.0000 | 0.5024 | 3.7751 | 2.0279 |
| HR | 0.1869 | -0.007189036 | -0.0176728 | 0.2201 | 3,228,418,131.0200 | 1,195,412,271.5100 | 2,163.0000 | 0.3647 | 3.5258 | 0.7275 |
| Gold Mantis | 1.4979 | -0.076037039 | -0.0204127 | 0.5814 | 81,980,893.4900 | 11,746,201,896.2600 | 17,254.0000 | 0.3720 | 14.7387 | 119.7324 |
| China Haisum | 1.1027 | -0.031027778 | -0.0123042 | 0.5380 | 6,236,965.1700 | 865,132,761.4300 | 4,866.0000 | 0.4392 | 8.2171 | 7.3659 |
| ZSSF | 2.1781 | 3.222407249 | 0.00592951 | 0.1704 | 1,507,295,729.1800 | 3,203,762,632.5200 | 5,338.0000 | 0.4527 | 2.5033 | 1.3336 |
| DHC | 2.1894 | -0.016721094 | -0.0442083 | 0.2987 | 111,023,874.8400 | 1,063,320,976.5600 | 1,228.0000 | 0.2367 | 7.1880 | 1.0624 |
| Yanhua Smartech | 7.4905 | 0.032495685 | 0.09555043 | 0.4223 | 30,815,570.9300 | 421,794,608.2400 | 965.0000 | 0.1880 | 2.7029 | 0.9849 |
| BXLQ | 18.0710 | 0.033133378 | -0.0403493 | 0.2801 | 2,244,834,378.2900 | 1,922,280,649.5300 | 4,243.0000 | 0.2875 | 3.4394 | 0.9893 |
| Orient Landscape | 6.2741 | -0.056637673 | 0.01635369 | 0.0520 | 511,052,225.2000 | 9,231,922,280.3000 | 3,388.0000 | 0.0637 | 1.4456 | 0.1605 |
| HONGTAO | 6.9357 | -0.012386027 | -0.0112563 | 0.1689 | 2,244,834,378.2900 | 6,471,907,314.3600 | 1,927.0000 | 0.2141 | 1.2338 | 58.4279 |
| YASHA | 2.5072 | 0.011292236 | -0.00772 | 0.2358 | 511,052,225.2000 | 4,049,929,734.6600 | 6,847.0000 | 0.2199 | 4.9227 | 1.7233 |
| PALM | 1.6554 | -0.693799976 | -0.4212262 | 0.0976 | 2,244,834,378.2900 | 1,719,541,394.2300 | 1,269.0000 | 0.1521 | 3.3949 | 0.3908 |
| Sanwei | 3.2824 | 0.3936294 | -0.0149624 | 1.8386 | 511,052,225.2000 | 339,925,208.4200 | 448.0000 | 0.1415 | 1.3932 | 1.8978 |
| GRANDLAND | 3.7039 | -0.01305025 | -0.0234556 | 0.3726 | 2,244,834,378.2900 | 4,296,947,810.8200 | 3,999.0000 | 0.2102 | 33.9549 | 3.7282 |
| HOLU | 5.7906 | 0.019362093 | 0.068308 | 0.1249 | 511,052,225.2000 | 1,672,567,404.6000 | 11,113.0000 | 0.5906 | 1.6541 | 0.9032 |
| ZHCGE | 7.4487 | -0.015573826 | -0.0483308 | 0.2041 | 2,244,834,378.2900 | 3,303,322,497.2200 | 2,005.0000 | 0.3090 | 1.6601 | 1.4566 |
| QDDFTT | 33.5102 | -0.002144385 | -0.0198374 | 0.7473 | 511,052,225.2000 | 573,772,518.9100 | 1,912.0000 | 0.3324 | 0.4786 | 0.7852 |
| Ruihe | 0.5124 | -0.017724565 | -0.013634 | 0.5309 | 248,954,333.7600 | 436,856,421.2400 | 699.0000 | 0.3138 | 1.8953 | 4.5931 |
| CDR&B | 0.0314 | 0.007294642 | -0.0219738 | 0.2847 | 44,747,165.4800 | 634,441,292.1200 | 690.0000 | 0.2720 | 21.0057 | 1.6193 |
| Pbland | 11.1643 | 0.025202901 | -0.0153977 | 0.4875 | 508,034,615.7000 | 1,480,409,302.0900 | 2,179.0000 | 0.1710 | 3.5585 | 0.6217 |

| Continued Table 6 Original data of indicators in the second quarter of 2020 | | | | | | | | | | |
| --- | --- | --- | --- | --- | --- | --- | --- | --- | --- | --- |
| Enterprises | Accounts receivable turnover | Operating gross profit margin | Return on equity | Net profit margin on sales | Return on assets | Price/book value ratio (PB) | Price earning (PE) ratio | Asset-liability ratio | Current ratio | Quick ratio |
| BAUING | 0.5350 | 16.6448 | 2.5396 | 3.8228 | 2.1668 | 1.6091 | 32.1704 | 62.5046 | 1.5178 | 1.5077 |
| CAMC | 0.7191 | 18.2048 | 0.7881 | 2.1519 | 0.4683 | 0.8332 | 53.0459 | 47.6748 | 1.8325 | 1.4012 |
| GHE | 1.4837 | 11.4771 | 3.3098 | 2.0332 | 1.7231 | 1.0615 | 16.1296 | 87.2707 | 0.7913 | 0.7395 |
| ZCT | 4.2387 | 6.8413 | 3.4855 | 2.0526 | 1.4839 | 0.9097 | 13.0132 | 74.1345 | 1.2733 | 1.2169 |
| HR | 3.5698 | 10.1577 | 4.8516 | 3.5761 | 1.4950 | 1.4591 | 15.2554 | 77.2817 | 1.1608 | 0.8376 |
| Gold Mantis | 0.7831 | 16.9487 | 6.5133 | 7.4411 | 3.0457 | 1.3408 | 10.4863 | 60.3701 | 1.5315 | 1.5280 |
| China Haisum | 2.2065 | 5.3926 | -4.8871 | -3.6327 | -1.2986 | 2.2562 | -22.2479 | 72.3415 | 1.2807 | 1.2788 |
| ZSSF | 1.2796 | 13.9728 | 5.4562 | 5.7238 | 2.5954 | 1.7186 | 16.0126 | 62.5526 | 1.1743 | 0.9861 |
| DHC | 1.2121 | 19.2167 | 4.3373 | 7.9858 | 1.6261 | 1.5775 | 18.3562 | 67.6149 | 1.3101 | 1.2830 |
| Yanhua Smartech | 0.6251 | 14.3395 | -1.8442 | -6.9028 | -0.8661 | 3.6542 | -98.1609 | 48.0790 | 1.5635 | 1.5290 |
| BXLQ | 1.8675 | 12.8577 | 0.9221 | 0.8853 | 0.6157 | 1.8970 | 124.1894 | 86.4885 | 1.1144 | 0.9189 |
| Orient Landscape | 0.1883 | 25.4120 | -1.5240 | -10.0903 | 0.3979 | 1.0615 | -34.5318 | 69.9093 | 1.2161 | 1.1939 |
| HONGTAO | 0.2932 | 19.0678 | 0.5115 | 1.8139 | 0.9519 | 1.2445 | 119.0857 | 69.3264 | 1.3731 | 1.3688 |
| YASHA | 0.4933 | 15.4625 | 1.5133 | 3.2304 | 0.8826 | 1.6928 | 56.1787 | 59.5241 | 1.3918 | 1.2419 |
| PALM | 1.0510 | 16.9998 | 0.6168 | 0.4858 | 0.9975 | 0.9712 | 78.8811 | 71.1953 | 1.1497 | 1.0703 |
| Sanwei | 0.4957 | 23.3218 | 2.2835 | 13.0467 | 1.6026 | 1.8342 | 39.7921 | 17.9595 | 4.3198 | 4.1336 |
| GRANDLAND | 0.5256 | 14.5114 | 0.4351 | 0.4683 | 0.7029 | 0.7412 | 85.2488 | 69.3774 | 1.4125 | 1.3366 |
| HOLU | 2.9913 | 13.8954 | 3.7894 | 3.7743 | 2.2326 | 3.0364 | 40.4875 | 62.6263 | 1.1376 | 0.5014 |
| ZHCGE | 0.6665 | 19.2734 | 2.5790 | 5.1301 | 2.0193 | 1.5334 | 28.7769 | 55.7963 | 1.4890 | 1.4532 |
| QDDFTT | 2.1002 | 26.5630 | 2.1604 | 13.3951 | 1.9392 | 1.2399 | 28.7002 | 35.9484 | 1.5229 | 1.1030 |
| Ruihe | 0.9304 | 16.1338 | 2.2017 | 4.6105 | 1.7363 | 0.8717 | 20.0971 | 53.2180 | 1.4781 | 1.3966 |
| CDR&B | 1.7451 | 6.3981 | 1.4707 | 4.5557 | 1.3915 | 1.0484 | 35.9510 | 59.4344 | 1.3065 | 1.2894 |
| Pbland | 0.5990 | 9.5841 | -0.5379 | -2.2353 | 0.0390 | 1.0112 | -93.8010 | 42.8131 | 2.1358 | 1.9306 |

| Table 7 Original data of indicators in the third quarter of 2020 | | | | | | | | | | |
| --- | --- | --- | --- | --- | --- | --- | --- | --- | --- | --- |
| Enterprises | The ratio of intangible assets | The growth rate of intangible assets | Increasing rate of fixed assets | Cash ratio | Inventory | Accounts receivable | Number of employees | Current asset turnover | Turnover of fixed assets | Inventory turnover |
| BAUING | 2.7458 | -0.007881441 | -0.0321107 | 0.4550 | 66,428,214.6400 | 2,757,707,549.0600 | 2,071.0000 | 0.4091 | 87.1203 | 16.2182 |
| CAMC | 3.0343 | -0.047480966 | -0.0204706 | 0.7403 | 3,910,447,108.1800 | 4,556,763,140.8300 | 5,761.0000 | 0.2997 | 3.4084 | 1.0451 |
| GHE | 1.0356 | -0.006453109 | -0.0044999 | 0.1373 | 931,573,074.3800 | 4,753,884,746.9200 | 4,220.0000 | 0.7052 | 0.9492 | 3.6729 |
| ZCT | 1.7364 | -0.013715517 | 0.01169846 | 0.3079 | 1,304,655,527.6800 | 2,056,782,490.6700 | 6,968.0000 | 0.8291 | 6.4926 | 3.4703 |
| HR | 0.1899 | -0.007241093 | -0.0154787 | 0.2340 | 3,160,399,782.3500 | 1,188,983,025.7400 | 2,163.0000 | 0.5895 | 5.8766 | 1.2059 |
| Gold Mantis | 1.3415 | -0.027067727 | -0.003659 | 0.5957 | 74,878,195.4100 | 12,654,083,111.0400 | 17,254.0000 | 0.6019 | 24.6572 | 207.8307 |
| China Haisum | 1.0525 | -0.005114737 | -0.0138707 | 0.6313 | 6,737,264.2200 | 809,115,154.8700 | 4,866.0000 | 0.8163 | 15.4743 | 13.4408 |
| ZSSF | 8.5461 | -0.003967649 | -0.0295554 | 0.1819 | 1,572,057,784.3000 | 3,291,208,526.8000 | 5,338.0000 | 0.7092 | 4.0889 | 2.1338 |
| DHC | 1.9831 | -0.014289879 | -0.0151237 | 0.3922 | 134,043,983.6700 | 934,084,848.4800 | 1,228.0000 | 0.4801 | 15.5352 | 2.3924 |
| Yanhua Smartech | 7.7615 | -0.028315121 | -0.0226167 | 0.3839 | 46,561,482.6700 | 416,964,020.9600 | 965.0000 | 0.3099 | 4.4084 | 1.5204 |
| BXLQ | 15.4414 | -0.003983088 | -0.0940694 | 0.3632 | 2,368,227,619.9000 | 1,648,761,475.5800 | 4,243.0000 | 0.4827 | 6.2387 | 1.7110 |
| Orient Landscape | 6.1083 | 0.036900299 | 0.00587247 | 0.0413 | 524,564,078.6900 | 9,531,078,900.5300 | 3,388.0000 | 0.1588 | 3.6710 | 0.4251 |
| HONGTAO | 6.7753 | -0.010910099 | 0.04092184 | 0.1832 | 28,013,981.7500 | 6,347,669,681.8300 | 1,927.0000 | 0.3013 | 1.7083 | 83.5429 |
| YASHA | 2.4867 | -0.013517576 | -0.0040098 | 0.2140 | 1,901,217,300.7100 | 4,103,035,634.9400 | 6,847.0000 | 0.3910 | 8.8280 | 3.0414 |
| PALM | 0.5456 | -0.007247883 | 0.27939528 | 0.0599 | 424,788,840.2000 | 2,378,925,496.0300 | 1,269.0000 | 0.3174 | 7.0058 | 0.8673 |
| Sanwei | 4.0983 | -0.036786206 | -0.0132503 | 1.9931 | 62,540,877.9800 | 291,731,042.5700 | 448.0000 | 0.2439 | 2.4293 | 2.9452 |
| GRANDLAND | 3.6508 | -0.012312538 | -0.0236636 | 0.4614 | 1,039,514,234.0500 | 3,815,510,470.7000 | 3,999.0000 | 0.3866 | 64.6583 | 7.1070 |
| HOLU | 5.5349 | -0.033110831 | 0.09838653 | 0.1840 | 5,112,864,385.6900 | 1,762,297,646.3500 | 11,113.0000 | 1.0664 | 2.9878 | 1.6873 |
| ZHCGE | 6.8273 | -0.015936729 | -0.066592 | 0.2805 | 178,487,552.9200 | 3,627,063,467.3100 | 2,005.0000 | 0.4829 | 2.8203 | 2.3890 |
| QDDFTT | 34.0423 | -0.003291745 | -0.0478877 | 0.8314 | 1,030,471,517.5400 | 501,782,795.3300 | 1,912.0000 | 0.5188 | 0.7633 | 1.2680 |
| Ruihe | 0.4814 | -0.016517286 | -0.0157339 | 0.5334 | 265,835,699.5400 | 510,827,607.4200 | 699.0000 | 0.5295 | 3.4073 | 7.9352 |
| CDR&B | 0.0293 | -0.011210715 | -0.0163186 | 0.2807 | 54,477,274.9500 | 667,508,489.2000 | 690.0000 | 0.4088 | 30.1472 | 2.3636 |
| Pbland | 11.1736 | -0.027807381 | -0.0362591 | 0.4082 | 541,235,594.8900 | 1,279,396,642.2000 | 2,179.0000 | 0.3052 | 6.3875 | 1.0457 |

| Continued Table 7 Original data of indicators in the third quarter of 2020 | | | | | | | | | | |
| --- | --- | --- | --- | --- | --- | --- | --- | --- | --- | --- |
| Enterprises | Accounts receivable turnover | Operating gross profit margin | Return on equity | Net profit margin on sales | Return on assets | Price/book value ratio (PB) | Price earning (PE) ratio | Asset-liability ratio | Current ratio | Quick ratio |
| BAUING | 0.8964 | 15.8681 | 3.5336 | 3.3720 | 3.1103 | 1.3216 | 28.6004 | 63.1011 | 1.5049 | 1.4959 |
| CAMC | 1.1408 | 21.0821 | 1.2031 | 2.1491 | 1.0474 | 0.8466 | 52.4636 | 48.0900 | 1.8134 | 1.4079 |
| GHE | 2.1246 | 11.3958 | 5.3190 | 2.1722 | 2.5466 | 1.1442 | 16.3944 | 87.4573 | 0.7842 | 0.7245 |
| ZCT | 7.0355 | 7.0216 | 5.5501 | 2.0081 | 2.4230 | 0.9836 | 13.3944 | 75.6349 | 1.2483 | 1.1932 |
| HR | 5.9204 | 10.5347 | 7.4109 | 3.4322 | 2.3395 | 1.4385 | 14.9715 | 77.5084 | 1.1748 | 0.8690 |
| Gold Mantis | 1.2735 | 17.0184 | 10.8521 | 7.7294 | 5.1217 | 1.5799 | 11.3572 | 61.0067 | 1.5213 | 1.5183 |
| China Haisum | 4.2768 | 7.6802 | 1.2948 | 0.5298 | 0.4825 | 2.1278 | 122.5811 | 70.9808 | 1.3028 | 1.3008 |
| ZSSF | 2.0315 | 13.4244 | 6.9987 | 4.5571 | 3.3943 | 1.7012 | 18.6780 | 63.6089 | 1.1666 | 0.9812 |
| DHC | 2.7780 | 14.1542 | 6.8766 | 5.9840 | 2.4846 | 1.6516 | 18.4071 | 69.4978 | 1.2647 | 1.2363 |
| Yanhua Smartech | 1.0136 | 15.2674 | -1.4394 | -3.5379 | -0.6756 | 3.5809 | -185.2409 | 46.4147 | 1.5911 | 1.5355 |
| BXLQ | 3.4793 | 11.2320 | 1.5871 | 0.9020 | 0.9118 | 2.0386 | 116.9395 | 86.9731 | 1.1916 | 0.9835 |
| Orient Landscape | 0.4720 | 22.3552 | 0.1462 | 1.0727 | 1.3804 | 1.0115 | 518.8063 | 69.9703 | 1.2169 | 1.1950 |
| HONGTAO | 0.4184 | 16.9273 | -1.7408 | -1.2574 | 0.6519 | 1.2983 | -53.8546 | 70.7708 | 1.2845 | 1.2804 |
| YASHA | 0.8800 | 14.9442 | 3.0261 | 3.6857 | 1.7173 | 1.6216 | 40.6755 | 59.3367 | 1.3949 | 1.2404 |
| PALM | 1.8529 | 17.0163 | 1.0593 | 0.8353 | 1.7953 | 1.1833 | 83.9984 | 71.7374 | 1.0554 | 1.0165 |
| Sanwei | 0.9172 | 26.9487 | 3.8057 | 14.2983 | 3.0978 | 2.0891 | 41.1107 | 17.6987 | 4.3373 | 4.1186 |
| GRANDLAND | 1.0189 | 14.9442 | 2.8622 | 2.4353 | 2.1513 | 0.7426 | 19.7144 | 69.8127 | 1.4105 | 1.3406 |
| HOLU | 5.5294 | 13.3279 | 9.7546 | 5.2796 | 5.2540 | 4.1359 | 33.0918 | 62.5967 | 1.2289 | 0.5759 |
| ZHCGE | 1.0444 | 19.2773 | 4.1059 | 4.9814 | 3.2023 | 1.5280 | 27.2305 | 58.0610 | 1.7215 | 1.6812 |
| QDDFTT | 3.4827 | 26.1605 | 3.0170 | 12.0674 | 2.6857 | 1.2271 | 30.5278 | 35.0020 | 1.6084 | 1.1915 |
| Ruihe | 1.6207 | 16.1517 | 5.1424 | 5.7039 | 3.3993 | 0.8957 | 13.4481 | 55.7808 | 1.4455 | 1.3692 |
| CDR&B | 2.4121 | 3.2068 | 2.1035 | 4.4921 | 2.1677 | 1.0563 | 38.1383 | 58.6609 | 1.2244 | 1.2026 |
| Pbland | 1.1295 | 12.6804 | -0.2533 | -0.5482 | 0.4093 | 0.9967 | -294.6618 | 41.4381 | 2.1382 | 1.9136 |
